# Supplementary material for: Cancer-Specific Loss of p53 Leads to a Modulation of Myeloid and T Cell Responses
Source: Cell Rep. 2020 Jan 14;30(2):481–496.e6. doi: 10.1016/j.celrep.2019.12.028 (PMC6963783; doi:10.1016/j.celrep.2019.12.028)
Supplement: Document S1. Figures S1–S6 [file mmc1.pdf]

Cell Reports, Volume *30*

## Supplemental Information

# Cancer-Specific Loss of p53 Leads to a Modulation of Myeloid and T Cell Responses

**Julianna Blagih, Fabio Zani, Probir Chakravarty, Marc Hennequart, Steven Pilley, Sebastijan Hobor, Andreas K. Hock, Josephine B. Walton, Jennifer P. Morton, Eva Gronroos, Susan Mason, Ming Yang, Iain McNeish, Charles Swanton, Karen Blyth, and Karen H. Vousden**

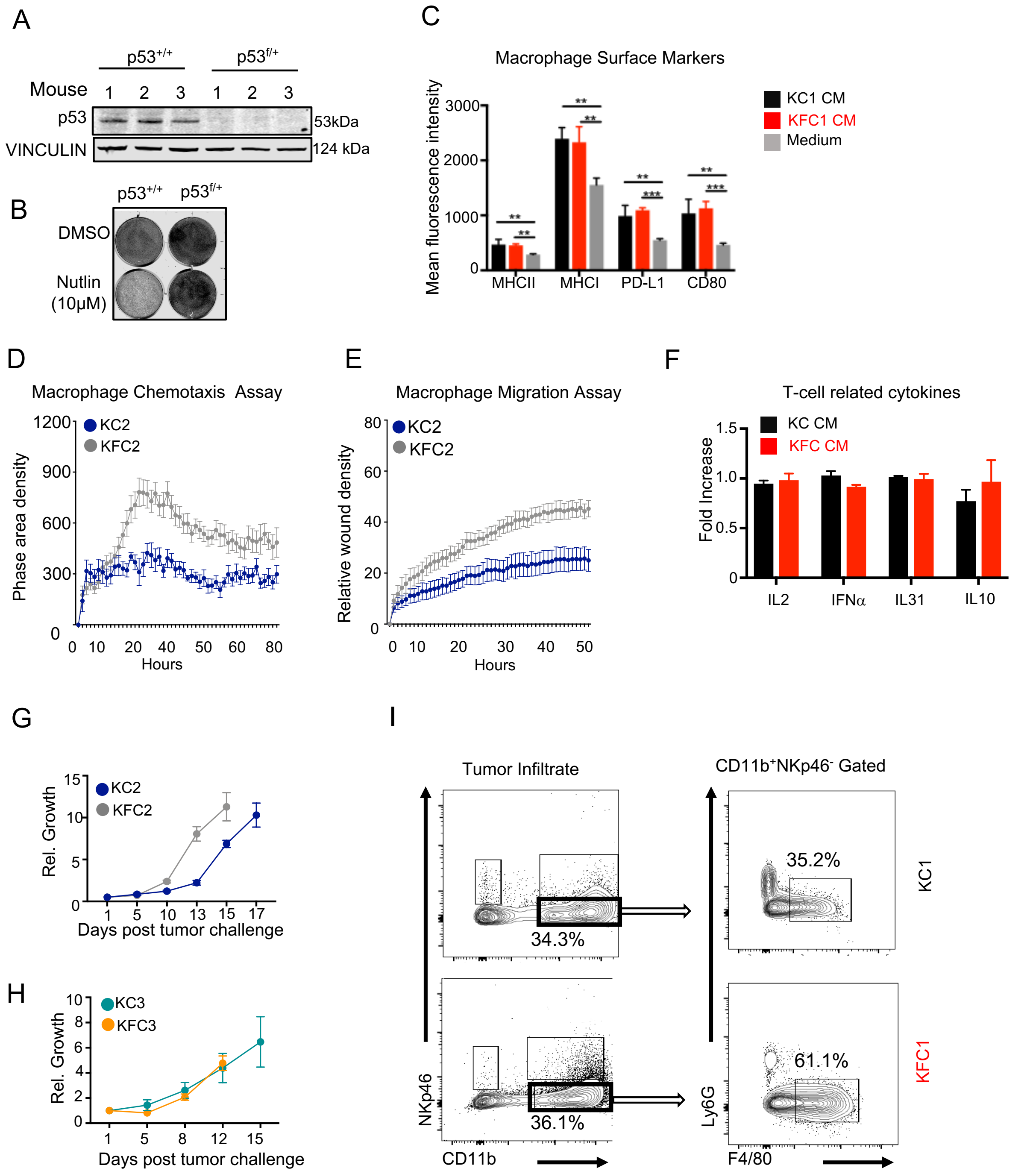


**Figure S1. Loss of p53 promotes macrophage migration and infiltration in athymic recipients, Related to Figure 1**

**A.** Immunoblot analysis of *Pdx1-cre; LSL-Kras*$^{G12D/+}$ (KC) and *Pdx1-cre; LSL-Kras*$^{G12D/+}$*; Trp53*$^{fl/+}$ (KFC) tumor-derived cell lines for p53 protein expression. VINCULIN was detected as a loading control. **B.** crystal violet assay of a *Trp53*$^{+/+}$ PDAC derived cell line and a *Trp53*$^{f/+}$ derived cell line in the presence of 10μM Nutlin for 48 hours. **C.** Bone marrow derived macrophages (BMDMs) were activated with medium (grey) or conditioned medium from the KC1 (black) or KFC1 (red) cell lines for 36 hours. Surface expression of MHCII, MHCI, PD-L1, and CD80 was assessed by flow cytometry. Graph represents one of three individual experiments and the mean ±SD of technical replicates (n=5). **D-E.**

Incucyte data from macrophage chemotaxis/migration assays with the mean represented as ±SD. (D) BMDM chemotaxis assay from conditioned medium from another set of PDAC-derived cell lines - the KC2 (blue) and KFC2 (grey) tumor-derived cell lines - measured over 80 hours. (E) BMDM migration-scratch wound assays looking at wound closure in the presence of conditioned medium from the KC2 (blue) and KFC2 (grey) cell lines over 50 hours measuring relative wound density. **F.** Luminex cytokine array performed on conditioned media from 3 independently derived KC (black) and KFC (red) cell lines. IL2, IFN$\alpha$, IL31, and IL10 were detected. Graph shows fold change from one KC cell line and the mean is represented as ±SEM of biological replicates. **G-H.** Subcutaneous growth of 2 independent p53 WT cell lines, KC2 (blue) and KC3 (teal), and p53-null cell lines, KFC2 (grey) and KFC3 (orange) in CD1$^{nu/nu}$ recipients with the mean represented as ±SEM of cohort sizes of n=5. All tumor derived cell lines were transduced with iRFP and growth was measured as relative growth to fluorescence at day 1 post injection by the Pearl Imager. **I.** Flow cytometry representative plot of intratumoral surface expression of Nkp46$^{+}$ and CD11b$^{+}$ immune cells (left) from individual tumors arising either from the KC1 or KFC1 cell line. Right representative plots are gated on CD11b$^{+}$Nkp46$^{-}$ populations and analyzed for F4/80 and Ly6G expression of tumors derived from the KC1 and KFC1 cell lines. Statistical p-values are denoted as *p<0.05 and ** p< p<0.01.

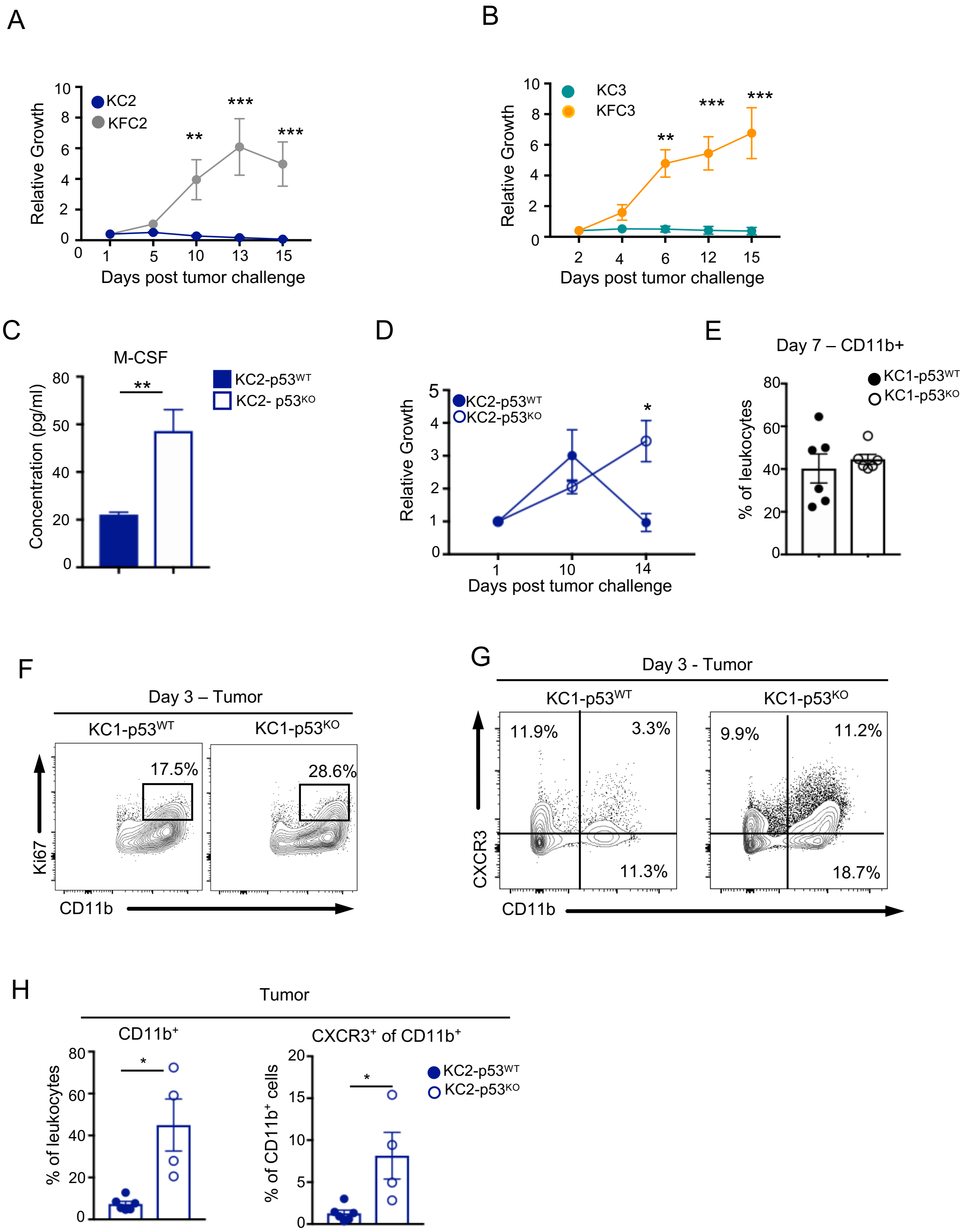


**Figure S2. p53 deletion retards tumor regression and increases myeloid infiltration, Related to Figure 2**

**A-B.** Real-time monitoring of tumor growth in MHC-mismatched hosts (FVB strain) by *in vivo* imaging using the Pearl imager with cohort sizes of n=5. Relative subcutaneous growth of a two independent sets of PDAC cell lines of both genotypes - (A) KC2 (blue) and KFC2 (grey) and (B) KC3 (teal) and KFC3 (orange) - transduced with iRFP. Relative growth is represented as the change in infrared fluorescence from day 1 post injection and the mean as ±SEM. **C.** Enzyme-linked immunosorbent assay (ELISA) for M-CSF in conditioned media from the isogenic cell lines, KC2- p53$^{WT}$ (blue) and CRISPR knockout, KC2-p53$^{KO}$ (open). The mean is represented as ±SD and the graph represents one of three independent

experiments. **D.** Monitoring tumor growth over 14 days by *in vivo* imaging of subcutaneous growth of isogenic cell lines – KC2-p53$^{WT}$ and CRISPR knockout for *Trp53*, KC2-p53$^{KO}$, in FVB MHC-mismatched recipients. Donor cells were transduced with iRFP and growth was made relative to infrared fluorescence day 1 post injection. Cohort sizes were n=5 and the mean measured as ±SEM. One-way ANOVA was used for statistical analysis. **E-G.** Flow cytometry analysis of individual tumor digests of the KC1-p53$^{WT}$ and KC1-p53$^{KO}$ cell lines injected into FVB recipients with cohort sizes of n=6-7 and the mean represented as ±SEM. (E) surface expression for CD11b$^{+}$ in tumor-immune infiltrates of KC1-p53$^{WT}$ and KC1-p53$^{KO}$ tumors at day 7 post injection. (F) Representative flow cytometry plot for tumor immune infiltrates expressing CD11b (X-axis) and Ki67 (Y-axis) of tumor digests at day 3 post injection. (G) Flow cytometry representative plots of KC1-p53$^{WT}$ and KC1-p53$^{KO}$ tumor immune infiltrates at day 3 looking at CD11b (x-axis) CXCR3 (y-axis) expression. **H.** Graphs show frequencies of CD11b$^{+}$ infiltrates and CD11b$^{+}$CXCR3$^{+}$ immune infiltrates at day 3 post tumor challenge in FVB mice (n=4-5) injected with the KC2-p53$^{WT}$ and KC2-p53$^{KO}$ cell lines with the mean represented as ±SEM. An unpaired Student's t-test was performed unless otherwise stated. Statistical p-values are *p<0.05 and ** p<0.01.

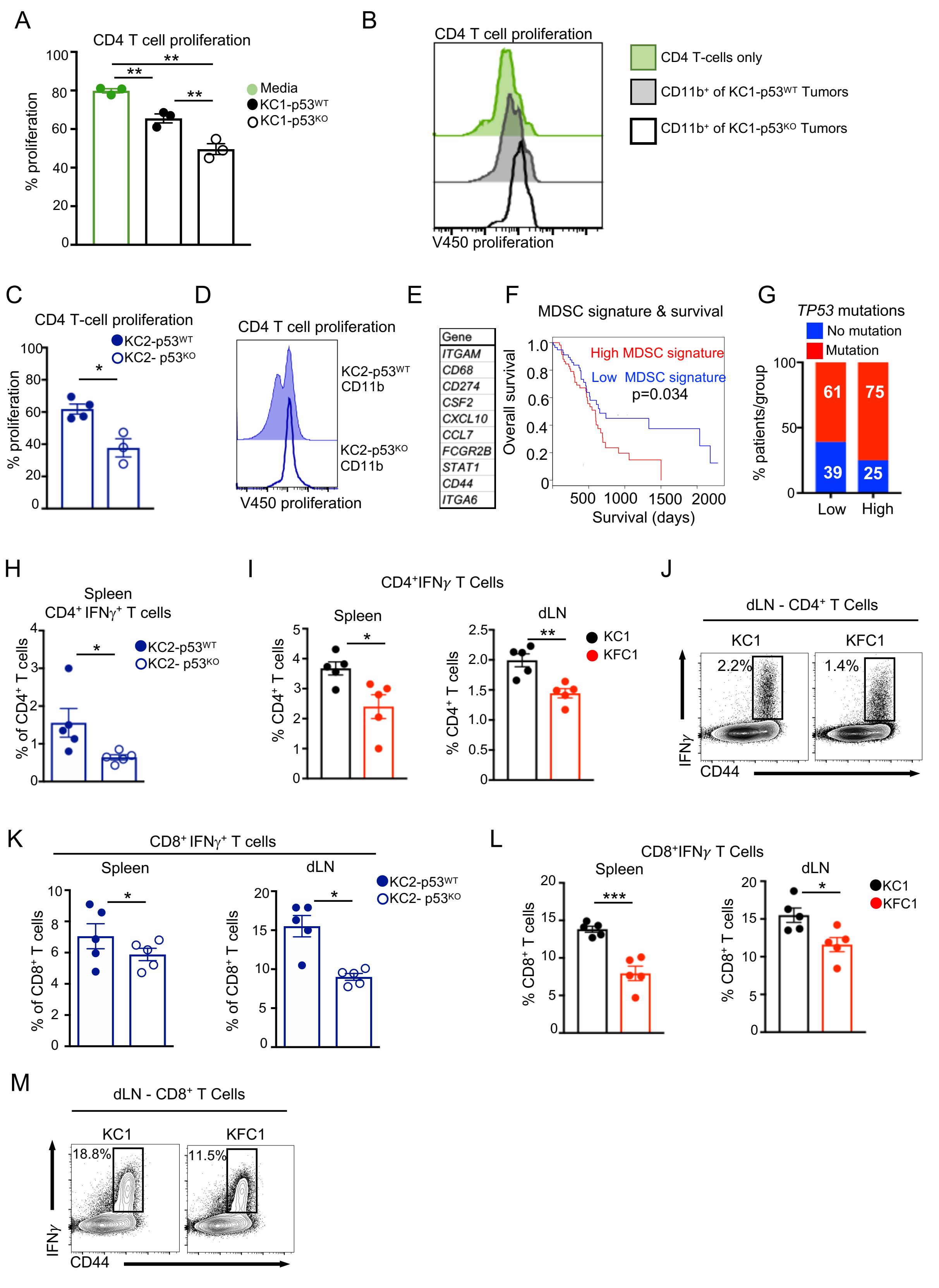


**Figure S3. Tumors null for p53 repurpose myeloid cells and dampen T-cell response, Related to Figure 3**

**A-D.** Tumor-associated CD11b$^+$ T cell suppression assays. Graph (A) and flow cytometry plots (B) represent CD4$^+$ T-cell proliferation either alone (green) or co-cultured with CD11b$^+$ cells isolated from KC1-p53$^{WT}$ (grey) or KC1-p53$^{KO}$ (white) tumors. Tukey's multiple comparison test was applied for statistical analysis and the mean is represented as ±SEM with each dot representing one CD11b$^+$ population derived from one individual tumor (n=3). (C) CD11b suppression assay with tumor-derived CD11b$^+$ cells from KC2-p53$^{WT}$ tumors (blue) and KC2-p53$^{KO}$ tumors (open circle) co-cultured with *in vitro* activated CD4$^+$ T cells (n=4-5). The mean is represented as ±SEM and an unpaired Student's t test was applied. (D) Representative T-cell proliferation blot of v450 proliferation dye dilution after 48 hours of co-incubation. **E-G.** Analysis of the pancreatic cancer patient cohort (PAAD) from The Cancer Genome Atlas (TCGA). (E) Myeloid derived suppressor cell (MDSC) gene signature used to define MDSC high (red) and low (blue) subsets in PAAD TCGA cohort. Sample size of 60 per high and low MDSC groups. (F) Survival curve of patients (top and bottom 60 patients) stratified on high (red) and low (blue) expression of the MDSC gene signature. (G) Percent of patients with *TP53* mutations (red) and no mutations (blue) in the high and low expression groups (n=60 per group). Statistical significance was determined by the log rank test and the mean as ±SEM. **H-M.** *Ex vivo* re-stimulation with PMA and ionomycin of splenocytes and dLN collected from FVB mice injected with one cell line per genotype at day 7 post tumor challenge followed by intracellular cytokine staining (ICS). Each point represents one mouse (cohorts size n=5) with the mean represented as ±SEM. (H) Flow cytometry analysis of spleens from mice bearing isogenic cell lines KC2-p53$^{WT}$ (blue circle) and KC2-p53$^{KO}$ (open circles) tumors stained by ICS for IFNγ. (I) Graphs show frequencies of IFNγ-producing CD4$^+$ cells in the spleen and dLN upon re-stimulation in KC1 (black) and KFC1 (red) tumor-bearing mice. (J) flow cytometry representative plot of *ex vivo* re-stimulated lymphocytes from tumor dLN from mice bearing KC1 or KFC1 tumors from each cell line showing CD44 expression against IFNγ signal. (K) Frequencies of CD8$^+$ T cell producing IFNγ upon *ex vivo* re-stimulation and ICS of splenocytes and dLN of FVB mice bearing isogenic cell lines KC2-p53$^{WT}$ (blue) and KC2-p53$^{KO}$ (open circle) tumors. (L) Bar graphs display frequencies of IFNγ-producing CD8$^+$ cells in the spleen and dLN upon *ex vivo* re-stimulation of KC1 and KFC1 tumor bearing FVB recipients. (M) Plots represent surface CD44 and IFNγ of gated CD8$^+$ T cells in the spleen of FVB mice harbouring KC1 or KFC1 tumors. An unpaired Student's t-test was applied where not specified. P-values range from *p<0.05, **p<0.01, ***p<0.001.

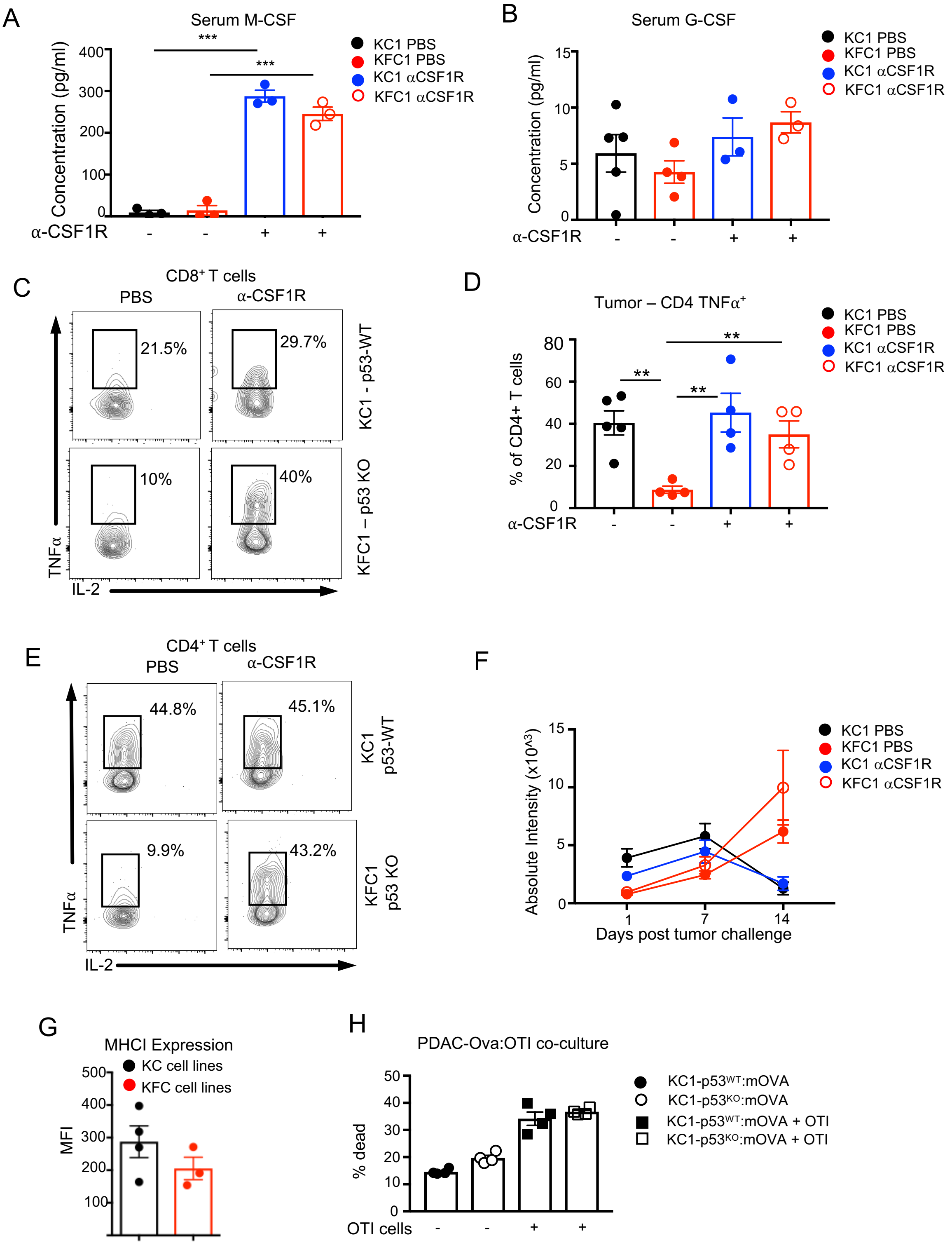


**Figure S4. αCSF1-R therapy relieves T-cell suppression in p53-deleted tumors independently of tumor growth and MHCI presentation, Related to Figure 4**

**A-B.** Serum from FVB mice injected with the KC1 and KFC1 cell lines either treated with PBS vehicle (black and red circles) or anti-CSF1-R blockade (blue and open red circles respectively) was collected and probed by ELISA for circulating (A) M-CSF and (B) G-CSF. Tukey's multiple comparison test was applied for statistical analysis and the mean is represented as ±SEM with sample sizes ranging from n=3-5. **C-E**. *Ex vivo* re-stimulation followed by ICS of tumor digests from tumors derived from the KC1

and KFC1 cell lines in FVB recipient mice treated with vehicle (PBS) or CSF1-R blockade. (C) Flow cytometry plots representing TNF$\alpha$ and IL2 staining of one individual tumor per cohort of infiltrating $CD8^+$ T cells. (D) Graph displays percentage of intratumoral $CD4^+$ T cells secreting TNF$\alpha$ from individual tumors from KC1 untreated (black), KFC1 untreated (red), KC1 treated (blue) and KFC1 treated (red open circles) tumor-bearing mice. Cohorts range from n=4-5 and the mean is represented as ± SEM with a Tukeys multiple comparison test applied. (E) Representative flow cytometry plot of intratumoral $CD4^+$ T cells expressing TNF$\alpha$ and IL2 **F.** Tumor growth kinetics show absolute fluorescence intensity of iRFP signal measured over 14 days by the Pearl imager with cohort sizes of n=5 per condition and the mean is represented as ±SEM. **G.** MHCI expression of 3 individual KFC and KC tumor-derived cell lines analysed by flow cytometry with the mean as ±SEM. Graph represents one of three independent experiments and an unpaired Student's t test was applied. **H.** PDAC isogenic cells ectopically expressing model antigen, ovalbumin, by retroviral introduction into KC1-$p53^{WT}$ (KC1-$p53^{WT}$:mOVA – black circles) and KC1-$p53^{KO}$ (KC1-$p53^{KO}$:mOVA – open circles). Pre-activated OTI cells with SIINFEKL peptide were co-cultured with isogenic cells expressing OVA (KC1-$p53^{WT}$:mOVA – black box and KC1-$p53^{KO}$:mOVA - open box). Cell viability was measured by propidium iodide (PI) staining and graphs show percentage of dead cells marked as $PI^{+ve}$. The mean is represented as ± SD and the graph represents one of three independent experiments. P values range from *p<0.05, **p<0.01, and ***p<0.001.

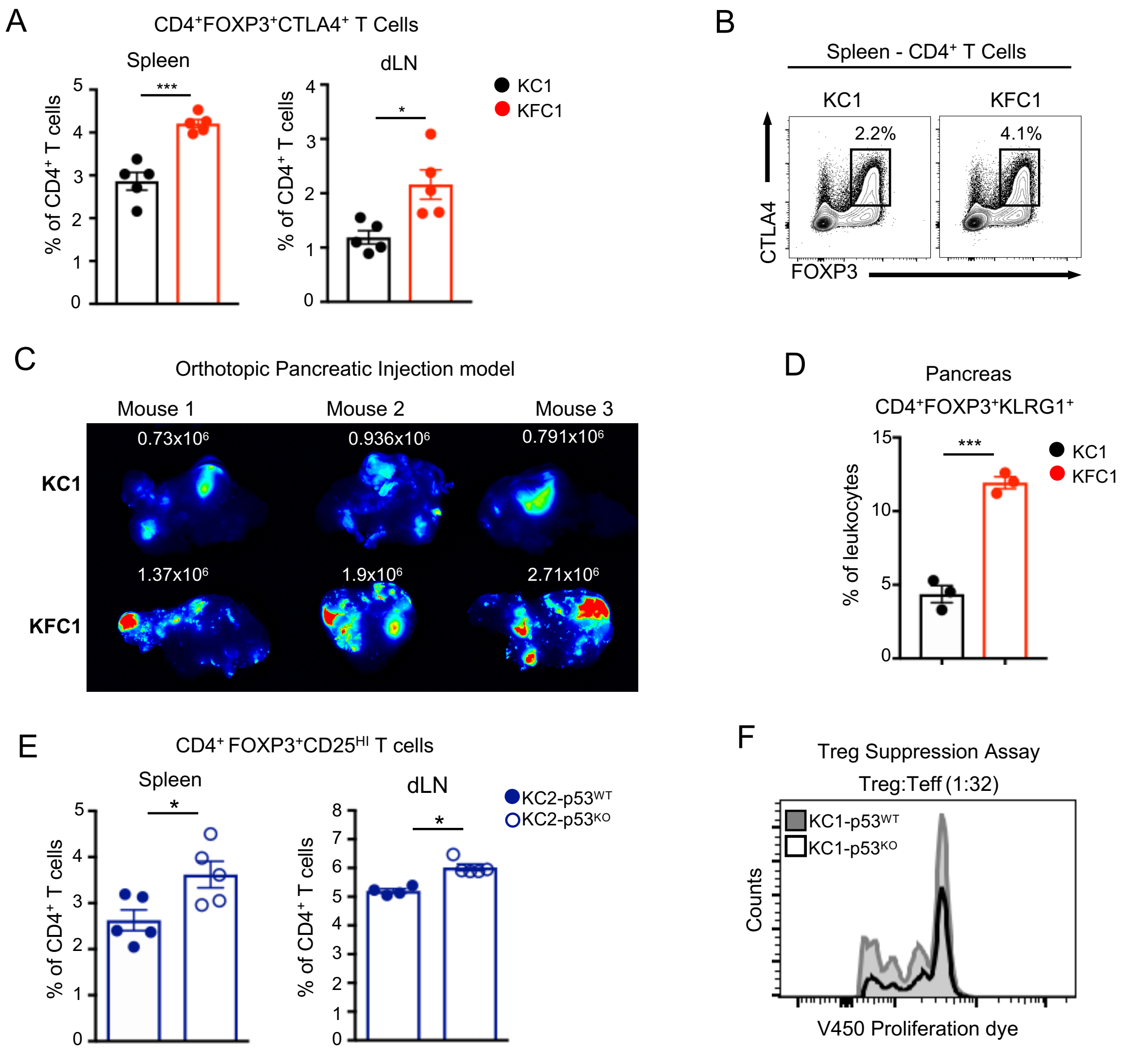


**Figure S5. Cancer-specific loss of p53 increases intra-tumoral and systemic regulatory T cells, Related to Figure 5**

**A-B.** Flow cytometry analysis for $CD4^+FOXP3^+CTLA\text{-}4^+$ Treg intracellular staining of splenocytes and dLN at 7 days of FVB mice injected with the KC1 (black) or KFC1 (red) cell lines (n=5) and the mean represented as ±SEM. (A) lymphocytes were gated on $CD4^+$ T cells and plotted for CTLA-4 and FOXP3 co-expression in the spleen and dLN. (B) Representative plot of one KC1 spleen and one KFC1 spleen gated on $CD4^+$ T cell populations and analyzed for CTLA-4 and FOXP3 co-expression. **C.** iRFP fluorescence analyzed *ex vivo* by the Pearl imager of orthotopic pancreatic injections into 3 FVB mice with the KC1 and KFC1 PDAC-derived cell lines 7 days post-surgery. **D.** Pancreatic infiltrating leukocytes gated on $CD4^+$T cells and analysed for CD4 and FOXP3 expression. The mean is represented as ±SEM. **E.** Surface and intracellular expression of $CD25^{hi}$ $FOXP3^+$ of splenic and lymphoid $CD4^+$ T cells in KC2-$p53^{WT}$ (blue) and KC2-$p53^{KO}$ FVB recipients (open). Cohort sizes were n=5 and the mean is represented as ±SEM. **F.** $CD4^+CD25^+$ T cells isolated and sorted from KC1- $p53^{WT}$ and KC1-$p53^{KO}$ tumor-bearing FVB mice and co-cultured with anti-CD3 (1µg/ml) and $CD4^+CD25^-$ T cells (Teff) stained with V450 proliferation dye. Representative flow cytometry plot of Teff proliferation co-cultured with KC1- $p53^{WT}$ Treg (grey shaded) and KC1-$p53^{KO}$ Tregs (black line) at a Treg:Teff ratio of 1:32. Unpaired Student's t-tests were performed and p values are *p<0.05, **p<0.01, ***p<0.001.

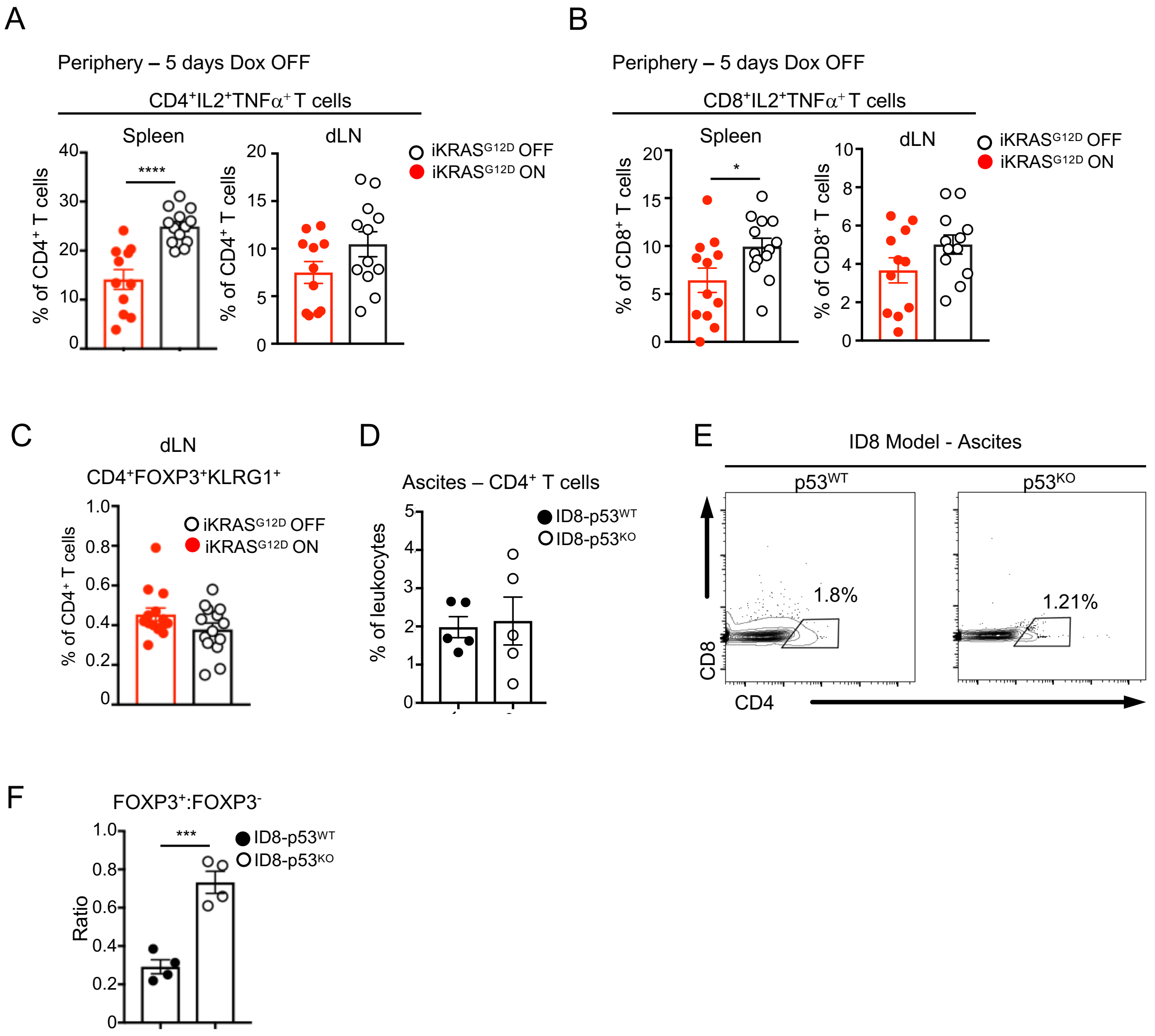


**Figure S6. *Kras* mutations coordinate with p53 loss for suppressing Teffector responses, but not Treg accumulation, Related to Figure 6**

**A-C**. Flow cytometry analysis at 5 days Dox OFF (open) and Dox ON (red) after 7 days post injection of iKRAS cell lines into FVB recipient mice. Cohort sizes were n=12-13 and each dot represents a single mouse with the mean represented as ± SEM. (A) Flow cytometry analysis of $CD4^+$ T cells of the spleen and dLN producing IL2 and TNFα upon *ex vivo* re-stimulation. (B) Flow cytometry analysis of $CD8^+$ T cells of the spleen and dLN producing IL2 and TNFα upon *ex vivo* re-stimulation. (C) Flow cytometry analysis of $CD4^+$ T cell populations expressing FOXP3 and KLRG1 in the dLN of FVB tumor bearing mice. **D-F.** Ascites of ID8 syngeneic (C57Bl6/J) model of ID8-$p53^{WT}$ (black) and ID8-$p53^{KO}$ (open circles) were analyzed by flow cytometry with sample sizes ranging from n=4-5 and the mean represented as ± SEM. (D) Graph shows $CD4^+$T cell frequencies of individual mice and the mean as ± SEM with a cohort size of n=5. (E) Flow cytometry representative plot for surface expression of CD4 (X-axis) and CD8 (Y-axis). (F) Graph shows the ratio of $FOXP3^+$ versus negative cells within the $CD4^+$ population in the ascites of ID8-$p53^{WT}$ (black) and ID8-$p53^{KO}$ donor cells (open circles). The mean is represented as ±SEM, n=4. Unpaired Student's t-test were performed for each graph. P-values range from *p<0.05, **p<0.01, ***p<0.001.
