# Supplementary material for: Cancer-Specific Loss of p53 Leads to a Modulation of Myeloid and T Cell Responses
Source: Cell Rep. 2020 Jan 14;30(2):481–496.e6. doi: 10.1016/j.celrep.2019.12.028 (PMC6963783; doi:10.1016/j.celrep.2019.12.028)
Supplement: Document S2. Article plus Supplemental Information [file mmc2.pdf]

Cell Reports

Article

Cancer-Specific Loss of p53 Leads to a Modulation of Myeloid and T Cell Responses

Graphical Abstract

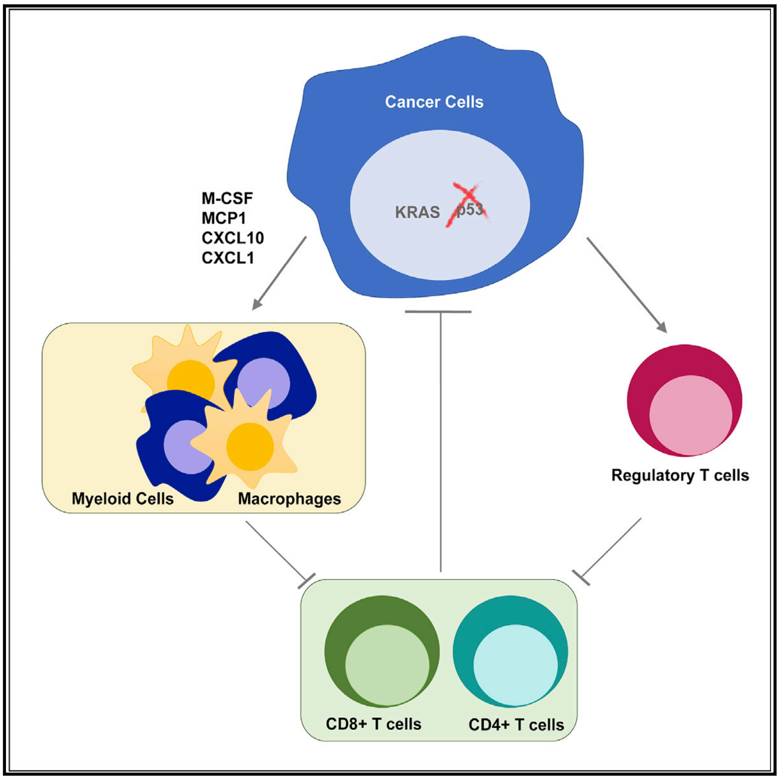

Highlights

- Tumor-specific loss of p53 delays tumor rejection in immune-competent hosts
- p53 loss increases myeloid infiltration through enhanced cytokine secretion
- The increase in Treg cells in response to loss of p53 is independent of Kras mutation
- Kras mutations coordinate with p53 loss to regulate myeloid recruitment

Authors

Julianna Blagih, Fabio Zani, Probir Chakravarty, ..., Charles Swanton, Karen Blyth, Karen H. Vousden

Correspondence

karen.vousden@crick.ac.uk

In Brief

TP53 is one of the most frequently mutated genes in cancer; however, its significance in controlling tumor-immune crosstalk is not fully understood. Blagih et al. highlight a key role for tumor-associated loss of p53, a common oncogenic event, in regulating myeloid and T cell responses.

Blagih et al., 2020, Cell Reports *30*, 481–496
January 14, 2020 © 2019 The Authors.
https://doi.org/10.1016/j.celrep.2019.12.028

Cell Reports
Article

OPEN ACCESS
CellPress

Cancer-Specific Loss of p53 Leads to a Modulation of Myeloid and T Cell Responses

Julianna Blagih,[1] Fabio Zani,[1] Probir Chakravarty,[1] Marc Hennequart,[1] Steven Pilley,[1] Sebastijan Hobor,[1] Andreas K. Hock,[2,4] Josephine B. Walton,[3] Jennifer P. Morton,[2,3] Eva Gronroos,[1] Susan Mason,[2] Ming Yang,[1] Iain McNeish,[3,5] Charles Swanton,[1] Karen Blyth,[2,3] and Karen H. Vousden[1,6,*]
[1]The Francis Crick Institute, 1 Midland Road, London NW1 1AT, UK
[2]Cancer Research UK Beatson Institute, Switchback Road, Glasgow G61 1BD, UK
[3]Institute of Cancer Sciences, University of Glasgow, Garscube Estate, Glasgow G61 1QH, UK
[4]Discovery Sciences, R&D BioPharmaceuticals, AstraZeneca, Cambridge CB4 0WG, UK
[5]Ovarian Cancer Action Research Centre, Department of Surgery and Cancer, Imperial College London, London W12 0NN, UK
[6]Lead Contact
*Correspondence: karen.vousden@crick.ac.uk
https://doi.org/10.1016/j.celrep.2019.12.028

SUMMARY

Loss of p53 function contributes to the development of many cancers. While cell-autonomous consequences of p53 mutation have been studied extensively, the role of p53 in regulating the anti-tumor immune response is still poorly understood. Here, we show that loss of p53 in cancer cells modulates the tumor-immune landscape to circumvent immune destruction. Deletion of p53 promotes the recruitment and instruction of suppressive myeloid CD11b$^+$ cells, in part through increased expression of CXCR3/CCR2-associated chemokines and macrophage colony-stimulating factor (M-CSF), and attenuates the CD4$^+$ T helper 1 (Th1) and CD8$^+$ T cell responses *in vivo*. p53-null tumors also show an accumulation of suppressive regulatory T (Treg) cells. Finally, we show that two key drivers of tumorigenesis, activation of KRAS and deletion of p53, cooperate to promote immune tolerance.

INTRODUCTION

There is strong evidence that cancer cells have the potential to be recognized by the immune system but that they can mobilize various mechanisms of immune evasion and escape, such as upregulation of immune checkpoint proteins to dampen T cell effector responses (Ribas and Wolchok, 2018). Current human studies have shown durable and complete responses to immune checkpoint blockades in a number of tumors; however, for reasons that are not completely clear, a sizable proportion of cancers fail to respond. It is apparent that the constellation of oncogenic events that leads to full neoplastic transformation can influence the effector function of the immune response in several ways. Oncogenic RAS can promote expression of various cytokines (Ancrile et al., 2008) that result in an inflammatory response, which is thought to promote cancer progression. RAS signaling also increases tumor cell expression of PD-L1 (Coelho et al., 2017), thereby suppressing activated T cells, and increases secretion of granulocyte-macrophage colony-stimulating factor (GM-CSF) to promote pancreatic neoplasia (Bayne et al., 2012; Pylayeva-Gupta et al., 2012). The co-activation of KRAS and MYC in lung tumors restructures macrophage and T cell responses in a CCL9 and interleukin-23 (IL-23)-dependent manner (Kortlever et al., 2017). Loss of PTEN, another common oncogenic event, results in resistance to PD-1 blockade in both melanoma and uterine leiomyosarcoma (George et al., 2017; Peng et al., 2016), while β-catenin signaling in melanoma was shown to limit T cell infiltration (Spranger et al., 2015).

p53 is best understood as a tumor suppressor (Hollstein et al., 1991). However, in the immune compartment, p53 also functions as a negative regulator of autoimmunity by supporting regulatory T (Treg) cells, through directly upregulating *Foxp3* and promoting STAT5 activity, and restricting STAT3 in the pro-inflammatory helper T cells (T helper 17 [Th17] cells) (Kawashima et al., 2013; Okuda et al., 2003; Park et al., 2013; Watanabe et al., 2014; Zhang et al., 2011). Expression of p53 in macrophages leads to both an inflammatory response through co-operation with nuclear factor κB (NF-κB) and an anti-inflammatory response through STAT1 inhibition (Lowe et al., 2014; Yoon et al., 2015; Zheng et al., 2005). In the context of cancer, activation of p53 in the tumor stromal compartment has been shown to promote a tumor-restricting immune response. Induction of p53 in hepatic stellate cells (HSCs) results in senescence and the senescent-associated-secretory phenotype (SASP) that drives M1-macrophage polarization and limits cancer progression (Lujambio et al., 2013). Conversely, HSCs lacking p53 induce the differentiation of macrophages toward the tumor-promoting M2 state (Lujambio et al., 2013). Stromal loss of p53 changes the cytokine secretion pattern to promote myeloid-derived suppressor cells (MDSCs), thereby accelerating tumor growth (Guo et al., 2013). Interestingly, activation of p53 in the tumor microenvironment using local injection of the MDM2 inhibitor Nutlin selectively eradicated tumors that were rich in leukocytes. This response was dependent on stromal-p53 expression (Guo et al., 2017). These studies show that p53 levels in the stroma shape the inflammatory responses that influence tumor progression.

This is an open access article under the CC BY license (http://creativecommons.org/licenses/by/4.0/).

Despite the clear role of p53 in immune regulation, relatively few studies have examined how p53 status of the cancer cells affects the immune response *in vivo*. Reactivation of p53 in established liver cancers induced senescence and SASP, which promoted polymorphonuclear (PMN) infiltration and tumor regression (Xue et al., 2007). Futher studies showed that the induction of p53-dependent senescence in hepatocellular carcinomas was accompanied by the elimination of the tumor cells through a mechanism dependent on natural killer (NK) cells (Iannello et al., 2013). *In silico* correlations between the retention of wild-type (WT) p53 expression and immune infiltration in breast and head and neck cancers have also been noted (Siemers et al., 2017). However, a recent study of a PTEN-driven prostate cancer model indicated that concomitant loss of p53 enhanced tumor infiltration of CD11b^{+}Gr1^{+} PMN cells. The recruitment of this myeloid population was through increased CXCL17 secretion by p53-null prostate cancer cells, and their role in promoting tumor development was associated with the expansion of immunosuppressive Treg cells (Bezzi et al., 2018). Similar findings were observed in mouse models of breast cancers, where loss of p53 increased frequencies of circulating and tumor neutrophils through unchecked WNT signaling, resulting in enhanced metastasis (Wellenstein et al., 2019).

In this study, we show that tumor-specific loss of p53 expression in both autochthonous lung and pancreatic tumor models correlates with changes in the tumor microenvironment. Using KRAS-driven pancreastumor-derived cancer cells as a model of p53 loss, we demonstrate that p53 deletion can promote immune tolerance through the recruitment of both myeloid cells and Treg cells. The enrichment of these suppressive populations results in enhanced protection of p53-null cancer cells from immune-mediated elimination. Furthermore, concomitant activation of KRAS and loss of p53 coordinate to promote immune tolerance.

RESULTS

Loss of *Trp53* Promotes Myeloid Recruitment in the Tumor Microenvironment

Tumor growth involves a complex interaction between stromal cells (of mesenchymal and immune origin) and cancer cells. Numerous studies have shown a role for macrophages in supporting cancer progression (Cassetta and Pollard, 2018; Noy and Pollard, 2014; Prenen and Mazzone, 2019; Qian and Pollard, 2010), and so we examined whether loss of p53 in autochthonous mouse models of pancreatic and lung cancers could influence myeloid cell recruitment to the tumor microenvironment (TME). Immunohistochemistry (IHC) sections were analyzed for F4/80^{+} immune cells in pancreatic tumors derived at equivalent endpoints from a pancreatic ductal adenocarcinoma cell (PDAC) model driven by pancreas-specific mutations in KRAS^{G12D} with either wild-type p53 (KC model; *Pdx1-cre; LSL-Kras*$^{G12D/+}$) or one floxed *Trp53* allele (KFC model; *Pdx1-cre; LSL-Kras*$^{G12D/+}$; *Trp53*$^{fl/+}$) (Tan et al., 2014) (Figure 1A, left). Tumors derived from KFC pancreatic tumors revealed increased macrophage F4/80^{+} staining (Figure 1A, right). We also examined an epidermal growth factor receptor (EGFR)-driven model of lung cancer with (ROSA26tTA-LSL-*EGFR*^{L858R}, EL) and without p53 (Rosa26tTA-LSL-*EGFR*^{L858R};*Trp53*$^{fl/fl}$, EFL). Tumors excised at similar endpoints were digested and stained for flow cytometry to detect CD11b^{+} and F4/80^{+} tumor immune infiltrates. Frequencies of CD11b^{+} myeloid-derived cells were significantly increased in EGFR-driven tumors null for p53, which were also enriched in CD11b^{+}F4/80^{+} macrophages (Figure 1B).

To gain insight into the interplay between macrophages and the p53 status of cancer cells, we isolated primary cancer cells from three independent KC and KFC tumors. KC-tumor-derived cells lines retained p53 expression and activity, showing growth inhibition in response to the p53 activator Nutlin, while cell lines derived from KFC tumors did not express p53 and were resistant to Nutlin treatment (Figures S1A and S1B). Previous studies have also shown that KFC tumors undergo loss of heterozygosity and become p53 null during tumor development (Tan et al., 2014). Reexpression of p53 in tumors can induce their ability to produce various inflammatory cytokines (Iannello et al., 2013), prompting us to examine whether conditioned media from the KC- and KFC-tumor derived cells could impact macrophage surface activation markers: major histocompatibility complex (MHC) class I, MHC class II, PD-L1, and CD80. In comparison to untreated bone-marrow-derived macrophages (BMDMs), conditioned media from all PDAC cell lines induced expression of all the activation markers to similar levels (Figure S1C), although this was not affected by p53 status. As both our autochthonous models (pancreas and lung) displayed a p53-associated change in macrophage infiltration, we examined the effect of conditioned medium on BMDM migration and chemotaxis. Interestingly, BMDMs exposed to conditioned media from KFC-derived cell lines displayed increased migration, demonstrated by enhanced wound healing and chemotaxis, compared to conditioned media from KC cell lines (Figures 1C, 1D, S1D, and S1E).

To explore the basis of this difference in the activity of the conditioned media, we analyzed cytokine secretion from three independently derived p53^{WT} KC and p53-null KFC tumor-derived cell lines. Out of the 35 cytokines tested by a cytokine Luminex array, 20 were detectable and only 5 (CCL11, CXCL1, CXCL5, CCL3, and macrophage colony-stimulating factor [M-CSF]) were elevated in the three KFC cell lines (Figure 1E). While these myeloid-attracting and differentiating cytokines were elevated, T-cell-associated cytokines were not dependent on p53 expression (Figure S1F). Taken together, these results indicate that the KFC tumors secrete cytokines that have a myeloid-macrophage stimulating effect.

To understand the consequences of the p53 status of our tumor-derived cell lines on the myeloid compartment *in vivo*, we used a T-cell-deficient CD1$^{nu/nu}$ subcutaneous tumor model. In order to track accurately and in an unbiased fashion *in vivo* tumor growth, KC and KFC cells were engineered to express near-infrared fluorescent protein (iRFP) (Hock et al., 2014; Shcherbakova and Verkhusha, 2013) (Figure 1F). While the initial GEMM KC and KFC tumors arise at different rates, cells derived from these tumors grew at similar rates in CD1$^{nu/nu}$ recipients, as assessed by *in vivo* real-time imaging of the tumors (Figures 1G, S1G, and S1H). Flow cytometry analysis of tumor digests at endpoint revealed no changes in total frequency of CD11b^{+} myeloid infiltration; however, CD11b^{+}F4/80^{+} macrophages were enhanced in KFC tumors, as seen in the autochthonous

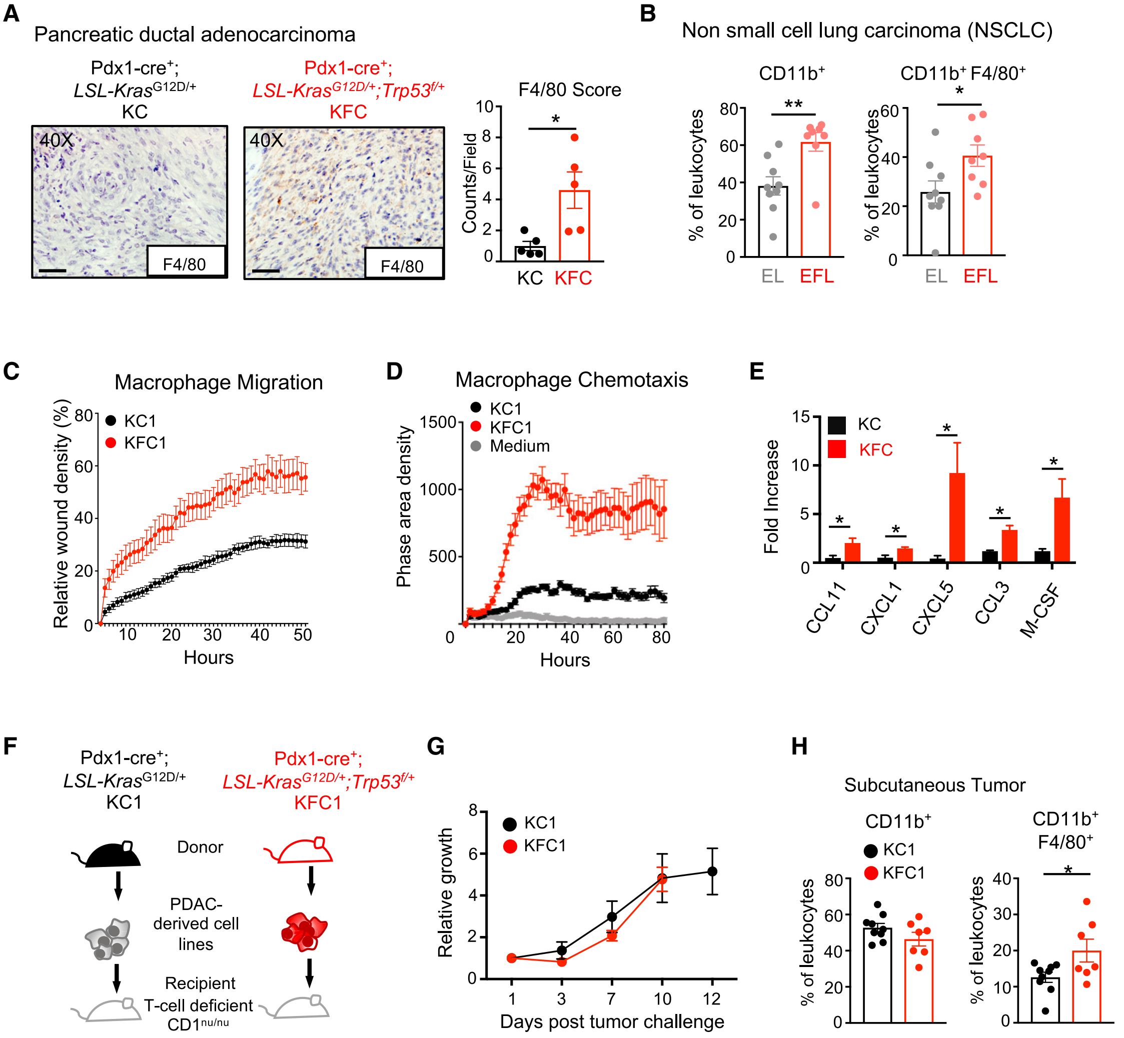

Figure 1. Cancer-Specific p53 Loss Increases Macrophage Infiltration, Chemokine Secretion, and Migration

(A) Immunohistochemical stain for F4/80 expression in pancreatic tumor sections from *Pdx1-Cre;Kras*$^{LSL-G12D/+}$ (KC) (left) and *Pdx1-Cre;Kras*$^{LSL-G12D/+}$*;Trp53*$^{fl/+}$ (KFC) (right) mice. F4/80$^+$ expression was evaluated based on color intensity per section. Scale bar at 1 μm. Each point on the graphs represents one mouse; cohort size n = 5, the means are represented as ± SEM.

(B) Lung tumors induced by adenoviral Cre were assessed by flow cytometry for CD11b$^+$ and F4/80$^+$ cell infiltrates from mice bearing the following genotypes: *ROSA26tTA-*^{LSL}*EGFR*^{L858R} (EL) (gray) and *Rosa26tTA-*^{LSL}*EGFR*^{L858R}*;Trp53*$^{fl/fl}$ (EFL) (red). Cohort sizes n = 8–9; the means are represented as ±SEM.

(C and D) Migration and chemotaxis assays using IncuCyte technology with bone-marrow-derived macrophages (BMDMs) cultured in the presence of conditioned media from PDAC-derived cell lines from KC1 (black) and KFC1 (red) tumors. The means are represented as ±SD of technical replicates (n = 6–8).

(C) Scratch-wound assay performed on BMDMs to measure wound closure.

(D) Chemotaxis assay of BMDMs migrating toward conditioned media of KC1 or KFC1 cancer cells.

(E) Luminex cytokine array performed on three independent KC and KFC cell lines derived from mouse PDACs. Values are represented as fold change in concentration compared to one of the PDAC-derived KC cell lines (KC1), and the means are represented as ± SEM.

(F) Schematic representation of the experimental design. Pancreatic derived cancer cell lines (KC1 and KFC1) were transduced with a near-infrared plasmid (iRFP) and subcutaneously injected into CD1$^{nu/nu}$ recipient mice.

(G) Growth curve represented as fold increase in iRFP fluorescence measured in real time by the Pearl imager over 12 days for KC1 (black circle) and KFC1 (red circle) cell lines injected into CD1$^{nu/nu}$ recipient mice (cohort size n = 5 per genotype). Two-way ANOVA was used for statistical analysis and the means are represented as ±SEM.

(H) Flow cytometry analysis of individual tumor digests for immune infiltrates expressing CD11b$^+$ and F4/80$^+$ surface markers. The means are represented as ±SEM, with cohort sizes n = 7–9.

Student's unpaired t test was performed when not otherwise indicated, and p values are $^*p < 0.05$ and $^{**}p < 0.01$. See also Figure S1.

OPEN ACCESS
CellPress

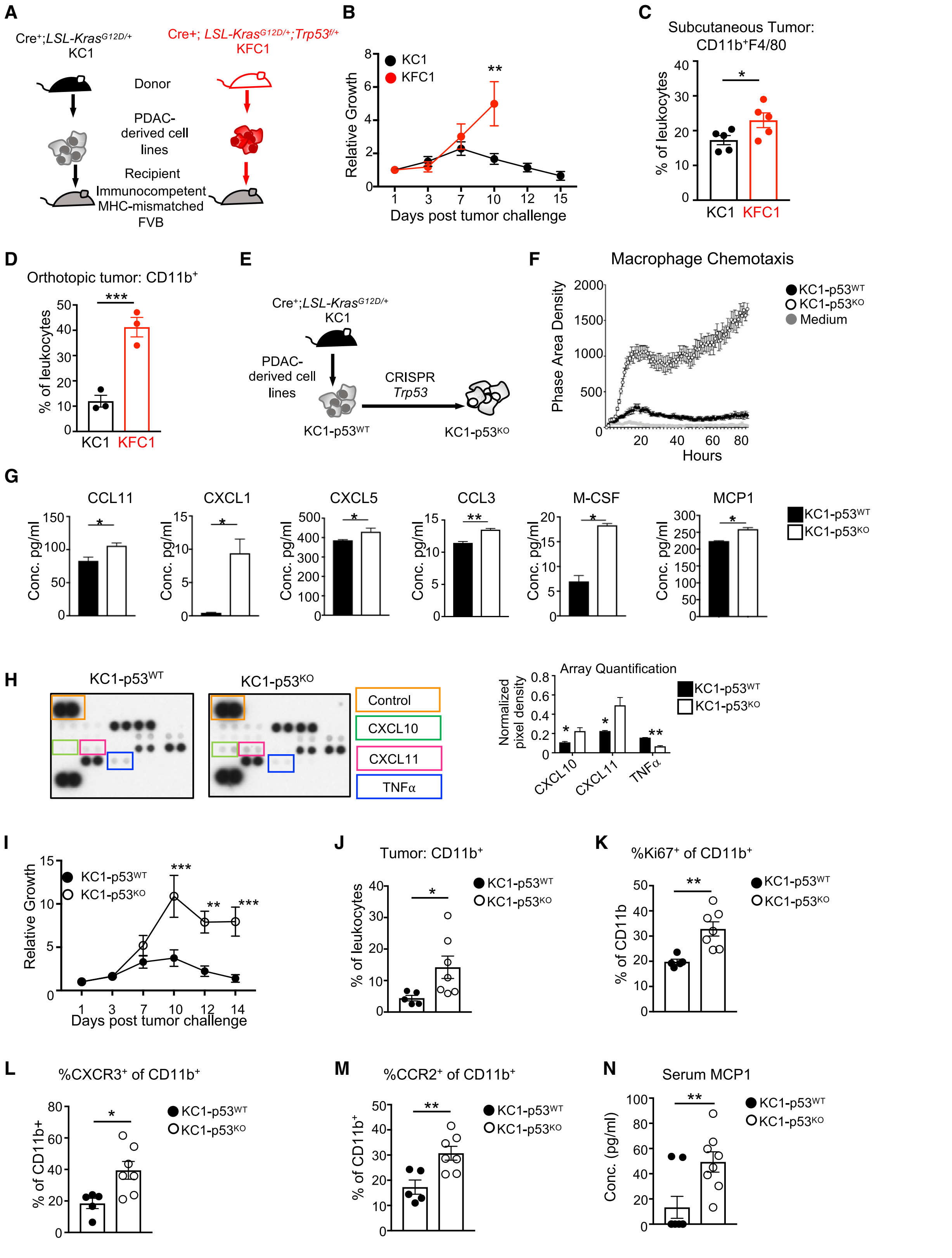

(legend on next page)

mouse models (Figures 1H and S1I). These results demonstrate that p53 ablation in the tumor can influence the TME independently of tumor growth.

p53 Deletion Delays Tumor Rejection and Promotes Myeloid-Associated Cytokines

Loss of p53 in the tumor-derived cell lines did not accelerate subcutaneous tumor growth in athymic recipients, despite the increase of $CD11b^+F4/80^+$ infiltrates in the TME. We therefore turned to examine the effect of p53 loss in an immunocompetent model. Since our tumor-derived cells originated from mice of a mixed background, we used a tumor rejection model that has been previously employed to explore contributions of tumor-associated genetic alterations on a complete immune response (Chang et al., 2015; Dunn et al., 2005). Mixed strain KC- and KFC-derived cells were injected subcutaneously into pure FVB recipient mice, an immunocompetent MHC-mismatched strain (Figure 2A). The $p53^{WT}$ KC1 cells followed the expected growth kinetics of initial expansion until day 7, followed by progressive rejection. Surprisingly, the p53-null KFC1 cells continued to grow until the point where ethical considerations determined the termination of the study (Figure 2B). A similar pattern of delayed rejection in p53-null cells was seen in two further independently derived KFC cells (Figures S2A and S2B). Further examination of the tumor digests taken from these mice at day 7 showed an increase in $F4/80^+$ macrophages in p53-null (KFC1) tumors (Figure 2C). In order to confirm that the p53-dependent effect on tumor-associated myeloid cells was not site specific, we performed pancreatic orthotopic injections of KC1 and KFC1 cell lines in FVB mice. In line with the genetic tumor models and the subcutaneous models (Figures 1A, 1H, and 2C), p53-null orthotopic tumors displayed increased myeloid infiltration (Figure 2D).

One potential caveat with tumors arising in genetically engineered mice is that additional genetic alterations acquired during tumor development may also contribute to tumor-immune interactions. To test directly the effect of p53 loss, we used gene editing to delete p53 from two KC-$p53^{WT}$ cell lines (KC1 and KC2), generating matched isogenic cell lines named KC1-$p53^{KO}$ and KC2-$p53^{KO}$ (Figure 2E). BMDM chemotaxis assays using conditioned media from KC1-$p53^{WT}$ and KC1-$p53^{KO}$ PDAC cell lines confirmed our previous findings that KFC-$p53^{KO}$ conditioned media enhanced BMDM migration (Figure 2F). ELISAs for CCL11, CXCL1, CXCL5, CCL3, M-CSF, and MCP1 (Figure 2G) showed that as seen in the tumor-derived KFC cell lines, p53 loss increased production of these cytokines (Figures 2G and S2C). Additionally, we performed an unbiased analysis of 62 cytokines in conditioned media from KC1-$p53^{WT}$ and KC1-$p53^{KO}$ cells, which further revealed increased CXCL10 and 11, and decreased tumor necrosis factor α (TNF-α) secretion (Figure 2H). Of note, CXCL10 and 11 uniquely bind to the CXCR3 receptor. Our results show that cancer cells deficient for p53 increase the production of chemokines involved in myeloid recruitment and macrophage differentiation.

Turning back to the *in vivo* rejection model, we found that the isogenic $p53^{KO}$ cells displayed delayed rejection kinetics, similar

Figure 2. Tumor Regression, Cytokine Production, and Myeloid Infiltration Are Influenced by p53

(A) Schematic representation of the experimental design. KC1 and KFC1 pancreatic-tumor-derived cell lines expressing iRFP were subcutaneously implanted into FVB, MHC-mismatched recipient mice.

(B) Growth curve of KC1 (black circle) and KFC1 (red circle) cell lines injected into five FVB recipient mice per genotype. Tumor growth is measured as an increase in iRFP signal compared to the original fluorescence count on day 1 using the Pearl imager. Two-way ANOVA was used for statistical analysis, and the means are represented as ±SEM.

(C) Individual tumors were harvested on day 7 post-injection and digested into single cells. Flow cytometry analysis was performed to measure $F4/80^+$ immune tumor infiltrates, and the means are represented as ±SEM.

(D) KC1 (black circle) and KFC1 (red circle) cells were orthotopically injected into the pancreas of FVB recipients (cohort size n = 3 per genotype). Graph shows percentage of pancreatic $CD11b^+$ infiltrates 7 days post-operation, and the means are represented as ±SEM.

(E) Schematic representation of the experimental design. KC1 PDAC cells were deleted for *Trp53* by CRISPR, generating an isogenic pair (KC1-$p53^{WT}$ and KC1-$p53^{KO}$).

(F) A chemotaxis assay performed on BMDMs migrating towards KC1-$p53^{WT}$ or KC1-$p53^{KO}$ conditioned medium, or culture medium. The extent of BMDM migration was calculated as phase area density of technical replicate wells (n = 8) and the means are represented as ±SD.

(G) Enzyme-linked absorbent assays (ELISAs) were performed with conditioned medium collected from KC1-$p53^{WT}$ (black) and KC1-$p53^{KO}$ (white) cells. ELISAs from left to right are CCL11, CXCL1, CXCL5, CCL3, M-CSF, and MCP1. Concentration was measured as pg/mL and the means are represented as ±SD.

(H) Cytokine array of conditioned media from KC1-$p53^{WT}$ (left) and KC1-$p53^{KO}$ (right) cell lines. (Left) Boxed duplicate dots show changes in CXCL10 (green), CXCL11 (pink), TNF-α (blue), with a positive invariant control (orange). (Right) pixel quantification of each dot normalized to the positive control and the means are represented as ± SD.

(I) 6 mice were injected with either the KC1-$p53^{WT}$ (black circle) or the KC1-$p53^{KO}$ (open circle) cells. The growth curve was measured by *in vivo* imaging of iRFP using the Pearl imager and graph shows fold increase in fluorescence from day 1 post-injection. Two-way ANOVA was used for statistical analysis, and the means are represented as ± SEM.

(J–M) Tumors derived from KC1-$p53^{WT}$ (n = 5) or KC1-$p53^{KO}$ cells (n = 7) were digested into single-cells at day 3 post-injection. Digests were analyzed for infiltrating myeloid populations by flow cytometry. Graphs show tumors from individual mice, and the means are represented as ±SEM.

(J) Flow cytometry analysis showing frequencies of tumor-infiltrating $CD11b^+$ myeloid cells in mice harboring KC1-$p53^{WT}$ (black circles) or KC1-$p53^{KO}$ (open circles) tumors.

(K) Frequency of proliferating intratumoral $CD11b^+$ populations measured by Ki67 staining.

(L) Frequency of CXCR3 expression on infiltrating $CD11b^+$ cells.

(M) Percentage of $CD11b^+$ cells within tumor digests expressing surface CCR2.

(N) Serum collected at endpoint (day 14) from FVB mice injected with the KC1-$p53^{WT}$ cell line (n = 8) or the KC1-$p53^{KO}$ cell line (n = 8) and analyzed for circulating levels of MCP1 by ELISA. The means are represented as ±SEM.

Student's unpaired t test was applied to experiments unless otherwise indicated and p values are $^*p < 0.05$, $^{**}p < 0.01$. See also Figure S2.

OPEN ACCESS
CellPress

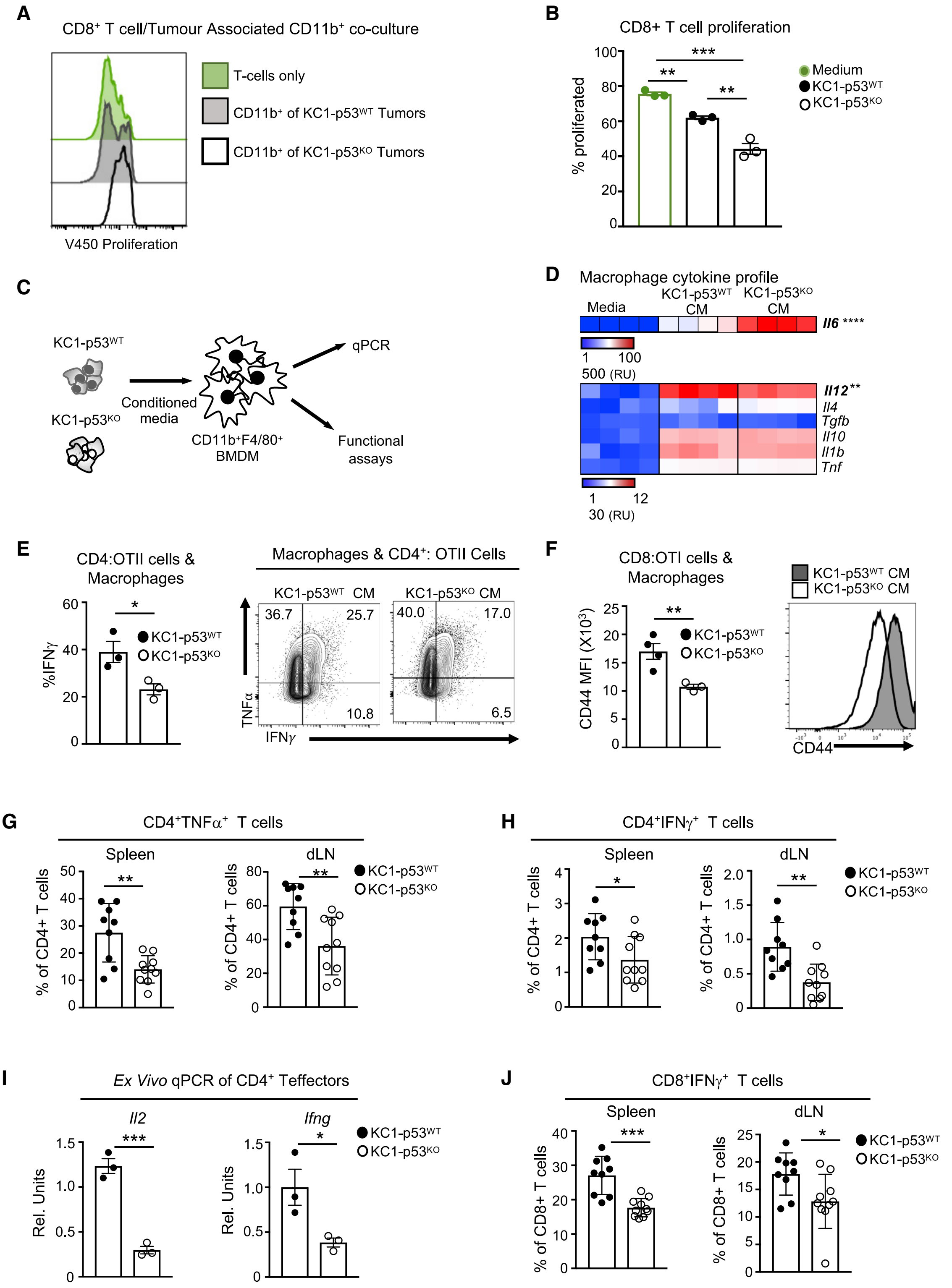

Figure 3. Tumoral Loss of p53 Promotes Suppressive Myeloid Lineages and Reduces T Cell Activation

(A) $CD11b^+$ cells isolated from individual KC1-$p53^{WT}$ (gray shade) and KC1-$p53^{KO}$ (black line) tumors 3 days post-injection and co-cultured with pre-activated $CD8^+$ T cells stained with V450 proliferation dye. Cells were analyzed after 48 h. T cell proliferation in the absence of $CD11b^+$ cells is denoted in green.

(legend continued on next page)

to PDAC cells that lost p53 during tumor development (Figures 2I and S2D). At an early time point (3 days post-injection), CD11b$^+$ myeloid cells were enriched in p53-deleted tumors in comparison to their parental p53^{WT} controls (Figure 2J), although this difference was lost by day 7 (Figure S2E). Next, we looked more closely for potential *in vivo* consequences of the changes in cytokine secretion detected *in vitro*. M-CSF is a key driver of myeloid differentiation and proliferation, and we detected a 2-fold increase of proliferating CD11b$^+$Ki67$^+$ myeloid cells in p53^{KO} tumors (Figures 2K and S2F), correlating with enhanced M-CSF production by these cells. We also probed for myeloid cells expressing CXCR3 (the receptor for CXCL9-11) and CCR2 (one of the receptors for CCL11 and uniquely a receptor for MCP1) (Martinelli et al., 2001) within our tumor digests. Interestingly, both CXCR3$^+$ and CCR2$^+$ infiltrating CD11b$^+$ myeloid cells were significantly enhanced within KC1-p53^{KO} tumors (Figures 2L, 2M, and S2G), correlating with increased production of the cytokines for these receptors by the p53-deleted tumor cells. We further confirmed an increase in intra-tumoral CD11b$^+$ cells expressing CXCR3 in the second isogenic tumor pair at day 3 (Figure S2H). Systemic cytokine changes were also observed in tumor-bearing mice. At endpoint (day 14 post-tumor challenge), mice harboring p53-null tumors displayed significantly increased serum levels of MCP1 (Figure 2N). In conclusion, data from both GEMM-derived cells and the isogenic pairs show that loss of p53 in tumor cells promotes the production of cytokines involved in myeloid recruitment and homeostasis.

p53-Null Tumors Reeducate Myeloid Cells to Attenuate T Cell Responses

Our observation that loss of p53 affects tumor growth in immunocompetent, but not athymic, mice indicated that an intact T cell response is required for the rejection of p53-expressing cells. To test whether the CD11b$^+$ cells found in the KC1-p53^{KO} tumors were immune suppressive, we isolated CD11b$^+$ cells from the tumors at day 3 and co-cultured them with preactivated T cells stained with the v450 proliferation dye. While the myeloid cells derived from both tumors attenuated T cell proliferation, the myeloid cells isolated from KC1-p53^{KO} tumors more robustly inhibited CD4$^+$ and CD8$^+$ T cell proliferation (Figures 3A, 3B, S3A, and S3B). These observations were confirmed in the second pair of isogenic cells (Figures S3C and S3D).

Cancer cells have been shown to influence the crosstalk between myeloid cells and T lymphocytes (Cooks et al., 2018), so we examined the effect of conditioned media from KC1-p53^{WT} and KC1-p53^{KO} cells on BMDM-dependent T-cell-activating functions (Figure 3C). Focusing on cytokines involved in CD4$^+$ T helper and CD8$^+$ cytotoxic T lymphocyte (CTL) differentiation, we found only two of the seven cytokines showed significantly changed mRNA expression in response to KC1-p53^{KO} conditioned media; namely, a strong increase in expression of *Il6,* a T helper 1 (Th1) cell antagonist, and a subtler decrease in expression of *Il12*, a Th1-cell- and CTL-promoting cytokine (Figure 3D). These changes predict that BMDMs conditioned by KC1-p53^{KO} media would show impaired polarization of CD4$^+$ Th1 cells and CTL activation (Athie-Morales et al., 2004; Dodge et al., 2003; Wu et al., 2015). To verify this hypothesis, we co-cultured BMDMs pulsed with the model antigen ovalbumin (OVA) with T cell receptor (TCR)-transgenic CD4$^+$ or CD8$^+$ T cells recognizing the OVA peptides (OTII [CD4$^+$] or OTI [CD8$^+$] T cells). BMDMs educated by KC1-p53^{KO} conditioned media and loaded with OVA peptide were less effective in differentiating Th1 cells, as demonstrated by reduced interferon-γ (IFN-γ)$^+$TNF-α^+-producing OTII CD4$^+$ T cells (Figure 3E). Furthermore, CTL activation was compromised by KC1-p53^{KO}-instructed BMDMs, as shown by the weaker expression of the activation marker

(B) Graph displays percent proliferation of CD8$^+$ T cells with tumor-derived CD11b$^+$ cells isolated from individual KC1-p53^{WT} (black) or KC1-p53^{KO} (white) tumors or in the absence of CD11b$^+$ cells (green). Each dot represents CD11b$^+$ cells derived from a pool of two tumors per genotype and means are represented as ±SEM.

(C) Schematic representation of the experimental design. BMDMs activated with conditioned media (CM) from KC1-p53^{WT} or KC1-p53^{KO} cells and screened for cytokine expression by qPCR and functional assays.

(D) mRNA expression of T-cell-polarizing cytokines expressed by BMDMs incubated in the presence of IMDM (medium), or KC1-p53^{WT}, or KC1-p53^{KO} conditioned media. Red indicates higher and blue lower expression levels, where each lane represents BMDMs derived from individual mice; the means are represented as ±SEM.

(E and F) Incubation of BMDMs with conditioned media, followed by a 2-h pulse of ovalbumin and co-cultured with CD4$^+$OTII or CD8$^+$OTI cells, respectively. Graphs show one of three experiments and display technical replicates (n = 3-4); means are represented as ±SD.

(E) OTII CD4$^+$ T cells restimulated with OVA 323-339 peptide after co-culture with BMDMs educated by KC1-p53^{WT} CM (closed circles) or KC1-p53^{KO} CM (open circles). Representative flow cytometry plot (right) of restimulated OTII T cells followed by intracellular cytokine staining (ICS) for IFN-γ and TNF-α after 4 days of differentiation.

(F) CD8$^+$OTI cells activated by BMDMs pulsed with OVA and measured by flow cytometry for surface expression of CD44, shown as mean fluorescence intensity (MFI). Left graph represents technical replicates for CD44 MFI, and on the right, the histogram shows CD44 surface expression.

(G–J) Analysis of CD4$^+$ and CD8$^+$ T cells in the periphery of FVB recipients injected with KC1-p53^{WT} (black) or KC1-p53^{KO} (white) cell lines. Graphs show biological replicates and the means are represented as ±SEM.

(G and H) Intracellular cytokine flow cytometry analysis of spleen and draining lymph nodes of FVB mice bearing KC1-p53^{WT} and KC1-p53^{KO} tumors (n = 9–10 FVB per genotype), 7 days post injection. Graphs illustrate (G) CD4$^+$ T cells expressing TNF-α and (H) CD4$^+$ T cells producing IFN-γ upon *ex vivo* restimulation with PMA, ionomycin, and GolgiStop (n = 9–10 per genotype).

(I) CD4$^+$ T cells isolated from KC1-p53^{WT} and KC1-p53^{KO} tumor-bearing FVB mice and cell sorted for CD4$^+$ CD25$^-$ populations. qPCR was performed on RNA isolated from *ex vivo* sorted CD4$^+$ CD25$^-$ T cells and tested for *Il2* and *Ifng* mRNA. The means are represented as ±SEM, and each point represents two pooled mice (cohort size n = 6 per genotype).

(J) *Ex vivo* restimulation of spleen and dLN from tumor bearing FVB mice to detect CD8$^+$T cells producing IFN-γ. The means are represented as ±SEM (cohort size n = 9–10 per genotype).

Unpaired t tests were performed on all data except for multiple comparisons, where Tukeys's multiple comparisons test was used. p values are *p < 0.05, **p < 0.01, and ***p < 0.001. See also Figure S3.

OPEN ACCESS
CellPress

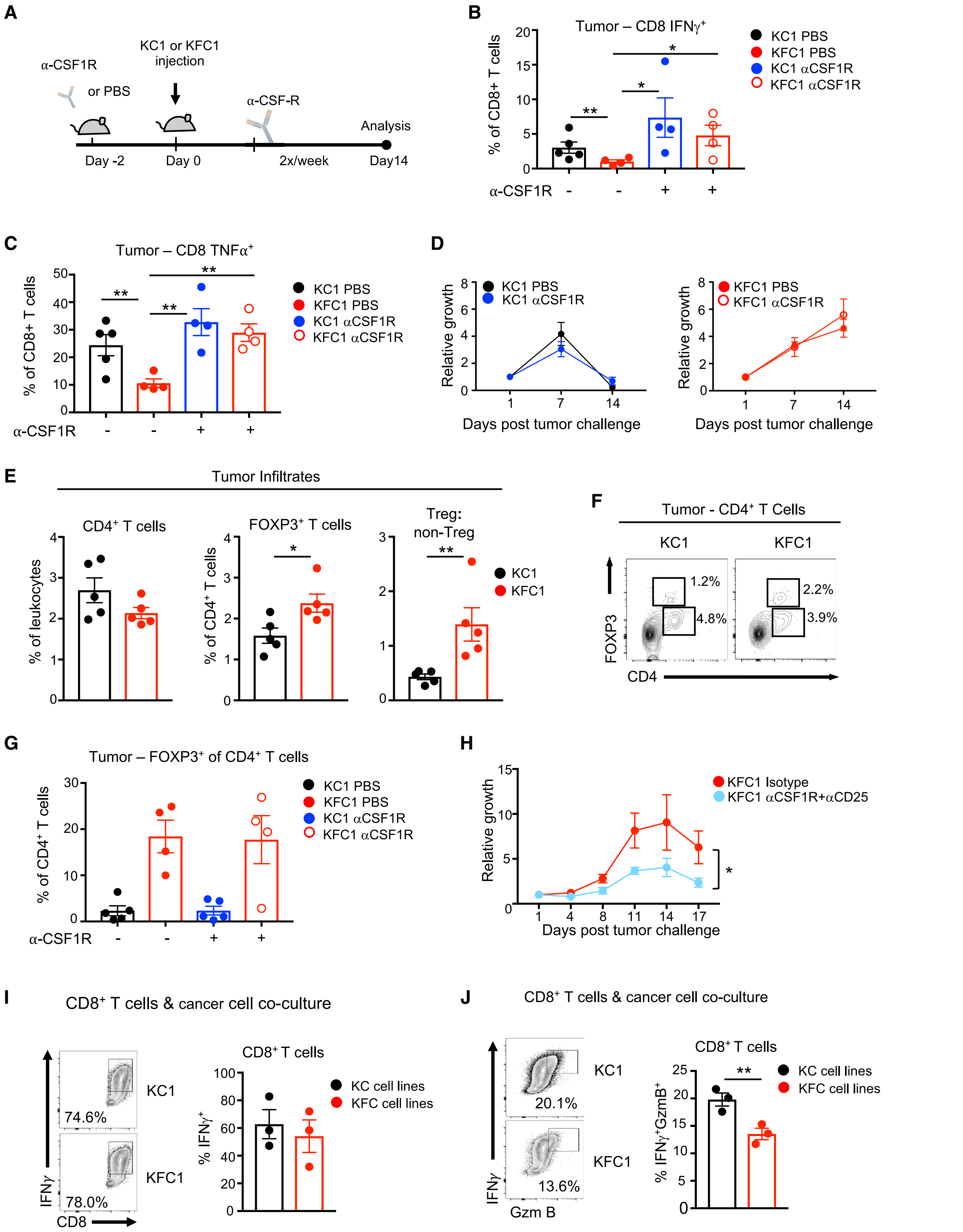

Figure 4. CSF1R Blockade Increases the Activation of Intratumoral T Cells in p53-Null Tumors but Depends on Treg Cell Depletion for Regression

(A) Scheme of CSF1R blockade administration and subcutaneous growth in FVB mice.

(legend continued on next page)

CD44 on $CD8^+$ OTI cells (Figure 3F). Led by our mouse data, we stratified pancreatic cancer patients from The Cancer Genome Atlas (TCGA) dataset according to a designed gene list based on classical MDSC markers, including *ITGAM* (CD11b) and *CXCL10* (Figure S3E). Patients with a high MDSC signature displayed statistically lower overall survival (Figure S3F). Interestingly, patients with a high MDSC gene signature trended toward an enrichment of *TP53* mutations compared to the low-MDSC-signature population (Figure S3G). Taken together, these data demonstrate that p53 deletion in cancer cells creates a tumor-promoting environment through remodeling myeloid-T cell crosstalk.

Encouraged by our *in vitro* and *ex vivo* results, we assessed T cell activity in mice bearing GEMM-derived or isogenic $p53^{WT}$ and p53-null tumors. *Ex vivo* restimulation of the spleen and tumor draining lymph node (dLN) on day 7, when tumors were of equivalent sizes, revealed a reduction in anti-tumor T cell responses (as measured by $CD4^+$ Th1 TNF-α^+ and IFN-γ^+ cells) in mice bearing p53-null tumors from either isogenic (Figures 3G, 3H, and S3H) or the GEMM-derived lines (Figures S3I and S3J). Furthermore, sorted $CD4^+CD25^-$ T cells from mice bearing KC1-$p53^{KO}$ tumors expressed less *Il2* and *Ifng* mRNA than $CD4^+CD25^-$T cells from KC1-$p53^{WT}$ recipients (Figure 3I), suggesting an overall lack of $CD4^+$ T cell activation. Impaired $CD8^+$ CTL IFN-γ^+ production further demonstrated weakened T cell responses in mice carrying p53-null tumors (Figures 3J and S3K–S3M). Overall, our data suggest that p53 deletion in cancer cells undermines T cell effector responses, at least in part through co-opting myeloid cell functions.

Combination Therapy of CSF1R and CD25 Blockade Attenuates p53-Null Cancer Cells

Our *in vitro* data suggested that the dampened T cell responses seen in mice bearing $p53^{KO}$ tumors may be a consequence of pro-tumorigenic myeloid cells responding to elevated M-CSF from p53-ablated cancer cells (Figure 2G). To test the contribution of M-CSF production by p53-null cells to the modulation of the T cell response *in vivo*, we treated immunocompetent mice challenged with either $p53^{WT}$ or p53-null tumor cells with neutralizing antibodies against CSF1R, the receptor for M-CSF (Figure 4A). CSF1R specificity was confirmed by showing the expected compensatory increase in serum M-CSF levels in treated mice (Bartocci et al., 1987), with no change in serum granulocyte colony-stimulating factor (G-CSF) (Figures S4A and S4B). Subcutaneous tumors were harvested at day 7, and effector function of infiltrating $CD4^+$ and $CD8^+$ T cells was assessed. Consistent with results from spleen and dLN of tumor-bearing mice, vehicle-treated, p53-null tumors contained less activated effector $CD4^+$ and $CD8^+$ T cells secreting IFN-γ and TNF-α (Figures 4B, 4C, and S4C–S4E). CSF1R blockade restimulated T cell function in p53-null tumors to levels seen in p53-WT tumors (Figures 4B, 4C, and S4C–S4E) but did not change the rate of rejection of tumors of either genotype (Figure 4D). Furthermore, T cell reactivation in p53-null tumors was irrespective of absolute tumor size, as confirmed by *in vivo* imaging of the tumors (Figure S4F). These data suggest that inhibition of M-CSF signaling that is induced by p53-deleted cells can reactivate T cell function within the tumor but fails to promote rejection when used as a monotherapy.

The failure of CSF1R blockade to promote tumor rejection prompted us to investigate other mechanisms of immune suppression that may reflect additional effects of tumor cells on T cell function. One essential immunosuppressive population that is at the epicenter of T cell immune tolerance are Treg cells (Sakaguchi et al., 2008), which are currently being targeted for cancer immunotherapies (Tanaka and Sakaguchi, 2017). Flow cytometry analysis of the original untreated tumors showed an accumulation of Treg cells within p53-null tumors with no changes in $CD4^+$ T cell frequencies (Figure 4E). Tumors deleted for p53 were biased toward Treg cells, as assessed by the Treg cell/non-Treg cell ratio of $CD4^+$ T cell infiltrates (Figures 4E and 4F). The inability of CSF1R blockade to reduce intra-tumoral Treg cell frequencies (Figure 4G) led us to perform a double blockade targeting both CSF1R and Treg cells using αCD25 to eradicate Treg cells. This method is well established to effectively deplete Treg cells (Onizuka et al., 1999; Setiady et al., 2010; Shimizu et al., 1999), although various studies have shown

(B and C) Isolated tumors at day 7 from mice injected with PBS (untreated) or CSF1R neutralizing antibody (treated), digested and *ex vivo* restimulated with PMA, ionomycin, and GoligStop. Each point represents one individual tumor (cohort size n = 4–5), and the means are represented as ±SEM. (B) $CD8^+$ IFN-γ^+ and (C) $CD8^+$ TNF-α^+ in KC1 control (black circles), KC1 treated (blue), KFC1 (red), and KFC1 treated (red open circles) mice.

(D) Subcutaneous growth of KC1 PDAC tumors treated with PBS (black line) or anti-CSF1R (blue) and KFC1 PDAC tumors treated with PBS (red) or anti-CSF1R (red open circle) in FVB recipients (cohort size n = 5 per condition). The means are represented as ±SEM.

(E) Tumors were processed and stained for $CD4^+$ infiltrates and expression of FOXP3. Graph on the right shows the ratio of Treg cell/non-Treg populations in tumor infiltrating $CD4^+$ cells. The means are represented as ±SEM with cohort sizes n = 5.

(F) Representative flow cytometry plots of CD4 and FOXP3 expressing T cells in individual tumors of each genotype.

(G) Flow cytometry analysis of tumor digests for $CD4^+$ $FOXP3^+$ T cell infiltration in tumors of untreated and treated mice, where each point represents one tumor. Means are represented as ±SEM, with sample sizes n = 4-5.

(H) Growth curve using *in vivo* imaging of the KFC1 tumors treated with isotype control (red) (n = 5) or with αCD25 treatment followed by αCSF1R treatment (blue) (n = 5) over 17 days post-tumor challenge. Graph shows relative growth compared to day 1 measured by *in vivo* fluorescence. Two-way ANOVA was used for statistical analysis and the means are represented as ±SEM.

(I and J) Flow cytometry analysis of *in-vitro*-activated $CD8^+$ T cells cocultured with individually derived PDAC cell lines.

(I) IFN-γ production of restimulated $CD8^+$ T cells with independent KC (KC1-3) or KFC (KFC1-3) PDAC cell lines shown as a representative flow cytometry plot (left) and as a graph showing biological replicates (right). Means are represented as ±SEM.

(J) IFN-γ^+GzmB$^+$-producing $CD8^+$ T cells assessed by restimulation and ICS. Representative flow cytometry plot of restimulated and stained $CD8^+$ T cells (left). Each dot on the graph (right) represents one co-culture of pre-activated $CD8^+$ T cells with KC (black) (KC1-3) or KFC (red) (KFC1-3) cell lines. The means are represented as ±SEM. Co-culture experiments represent one of three independent experiments.

An unpaired Student's t test was applied unless otherwise indicated. p values are $^*p < 0.05$ and $^{**}p < 0.01$. See also Figure S4.

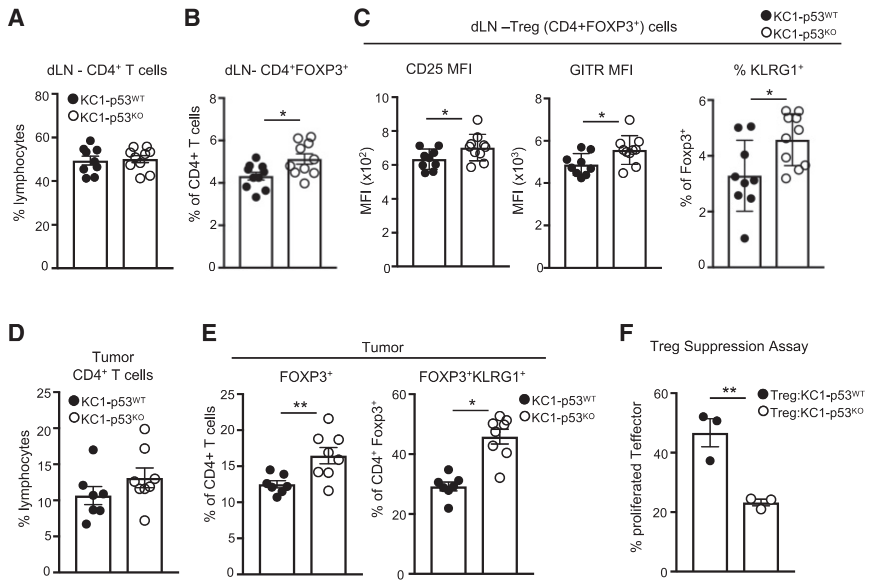

Figure 5. p53 Loss Increases Intratumoral and Systemic Suppressive Treg Cell Lineages

(A–E) Flow cytometry analysis of tumors from FVB mice injected with the isogenic cells lines KC1-p53^{WT} (black circles) and KC1-p53^{KO} (open circles) and analyzed 7 days post-injection. Cohort sizes n = 7–10 , the means are represented as ±SEM.

(A) CD4^{+} T cell frequencies in the dLN from KC1-p53^{WT} KC1-p53^{KO} recipients.

(B) Intracellular staining of FOXP3 and CD4 surface expression in T cells within the dLN.

(C) The mean fluorescence intensity (MFI) of CD25 and GITR and the frequency of KLRG1 surface expression within Treg cell populations in tumor dLN.

(D) Frequencies of CD4^{+} T cell infiltration in KC1-p53^{WT} KC1-p53^{KO} tumors.

(E) Intracellular staining for FOXP3 in tumor-infiltrating CD4^{+}T cells (left) and surface expression of KLRG1 within the CD4^{+}FOXP3^{+} T cell population (right).

(F) *Ex vivo* Treg suppression assay of sorted CD4^{+}CD25^{+} Treg cells from mice (cohort size n = 6) injected with KC1-p53^{WT} (black circles) and KC1-p53^{KO} (open circles) cell lines. Data show percentage of proliferating co-cultured CD4^{+}CD25^{-} T cells that were stained with v450 proliferation dye. Each point represents CD4^{+}CD25^{+} Treg cells from two pooled recipient FVB mice, and the means are represented as ±SEM.

An unpaired Student's t test was used for statistics with p values of *$p < 0.05$ and **$p < 0.01$. See also Figure S5.

that CD25 blockade alone does not effectively block the growth of established tumors (Arce Vargas et al., 2017; Onizuka et al., 1999). Using a combined treatment of αCD25 for 3 days prior to tumor challenge followed by an αCSF1R regime, we were able to attenuate the growth of the p53-null tumors in FVB recipients (Figure 4H). These intervention data suggest that immunotherapy for p53-compromised tumors requires targeting both the myeloid and Treg cell compartments for a positive outcome.

Finally, we examined the consequences of direct interactions between CD8^{+} cytotoxic T lymphocytes (CTLs) and cancer cells. CTL cytotoxic function is elicited by IFN-γ and granzyme B (GzmB) production and can be affected by cancer cells (Fischer et al., 2007). CTLs co-cultured with KC-p53^{WT} and KFC-p53^{KO} cells were equally capable of secreting IFN-γ (Figure 4I). However, co-culture with KFC-p53^{KO} cells clearly impaired CTL GzmB production upon restimulation (Figure 4J).

Tumor cells can evade CTL responses through downregulation of MHC class I surface expression (McGranahan et al., 2017), although this was not observed in the p53-null PDAC-tumor-derived cells (Figure S4G). In order to assess if loss of p53 in cancer cells influences their ability to present endogenous peptides, we introduced the model antigen, OVA, into the isogenic PDAC cell lines. Co-culturing of peptide-stimulated OTI cells with KC1p53^{WT}-mOVA and KC1p53^{KO}-mOVA increased cell death in both PDAC cell lines, as marked by increased propidium-iodide-positive cells. However, loss of p53 did not lead to an increased resistance to OTI killing by MHC class I (Figure S4H). Taken together, these results indicate that immune evasion in response to loss of p53 reflects both an accumulation of suppressive Treg cells and inhibition of T cell cytotoxic functions.

CancerCell-Associated Loss of p53 Selects for Suppressive Regulatory T Cell Lineages

Our observation that targeting Treg cells in parallel with CSF1R was critical in reducing tumor growth of p53-null cells prompted us to examine the effect of the p53 status of cancer cells on regulatory T cells more closely. Treg cells have different degrees of suppressive abilities, which can be phenotypically identified through surface expression of CLTA-4, KLRG1, and GITR and high expression of CD25 (Arpaia et al., 2015). Mice bearing KFC1 p53-null tumors displayed increased systemic presence of CD4^{+}FOXP3^{+}CTLA-4^{+} T cells in the spleen and dLN (Figures S5A and S5B), suggesting a greater suppressive immune response. To determine whether the skewing toward more suppressive Treg cells depended on the site of the tumor, we orthotopically implanted KC1 and the KFC1 cell lines into the pancreata of FVB recipients. Tumors of both genotypes were detected in the pancreas at day 7 (Figure S5C) with a strong accumulation of highly suppressive Treg KLRG1^{+} cells in p53-null pancreatic tumors (Figure S5D).

To validate the p53 specificity of these Treg cell responses, we turned again to the isogenic KC1-p53^{WT} and KC1-p53^{KO} cells. Identical CD4^{+} T cell frequencies were present in the tumor dLN of both KC1-p53^{WT} and KC1-p53^{KO} recipients (Figure 5A). However, the proportion of FOXP3^{+} (Treg) cells present within the CD4^{+} population was enhanced in mice bearing KC1-p53^{KO} cancer cells (Figure 5B). Phenotypic characterization of these Treg cells revealed an increase in the proportion expressing markers of suppression (high CD25, GITR, and KLRG1 expression) (Figures 5C and S5E). In addition, while p53 status did not affect total CD4^{+} T cell frequency in the tumor (Figure 5D), KC1-p53^{KO} tumors accumulated more FOXP3^{+} Treg cells, especially those with KRLG1 surface expression (Figure 5E).

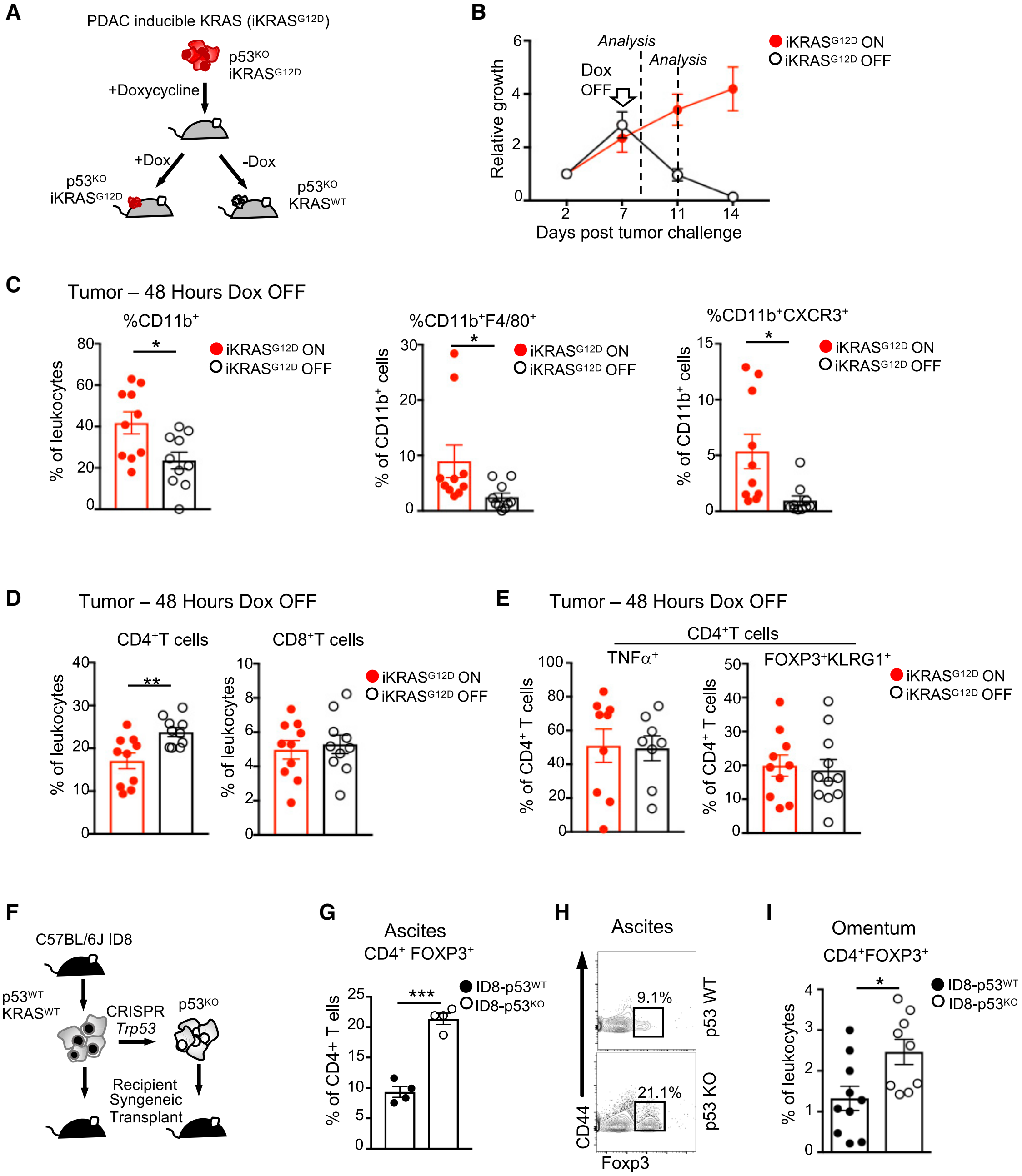

Figure 6. KRAS Coordinates with p53 Loss for the Recruitment of Myeloid, but Not Treg, Cells

(A) Schematic representation of the inducible $iKras^{G12D/+};Trp53^{f/f}$ PDAC cell model (iKRAS^{G12D}).

(B) Growth kinetics of the iKRAS^{G12D} cell line (red circles) expressing iRFP subcutaneously injected into FVB recipients. Doxycycline (Dox) was maintained (red circles) or removed at day 7 (open circles) to modulate mutant KRAS expression. Tumor growth was measured using *in vivo* imaging using the Pearl imager and expressed relative to initial iRFP fluorescence at day 1 post-injection. Cohort sizes n = 10 and the means are represented as ±SEM.

(C–E) Analysis of individual tumor infiltrates of myeloid and T cell subsets 48 h post-Dox withdrawal. Cohort sizes n = 8–12 and the means are represented as ±SEM.

(C) Analysis of CD11b$^+$, CD11b$^+$F4/80$^+$, and CD11b$^+$CXCR3$^+$ tumor-associated myeloid cells analyzed by flow cytometry (open circles Dox OFF and red circles Dox ON).

(D) Flow cytometry analysis of CD4$^+$ and CD8$^+$ T cell infiltrate frequencies.

(E) Analysis of intra-tumoral CD4$^+$ T cell post *ex vivo* restimulation for expression of TNF-α^+ (left) and CD4$^+$FOXP3$^+$KLRG1$^+$ (right).

(F) Schematic representation of experimental design. C57Bl6/J-derived ID8 ovarian cancer cell lines were deleted of *Trp53* using CRISPR/Cas9 and injected intraperitoneally into syngeneic recipients.

(G–I) Flow cytometry analysis of ascites and omentum at equivalent endpoints from mice bearing ID8-p53^{WT} (black circles) and ID8-p53^{KO} (open circles) tumors.

(legend continued on next page)

Consistent with these data, *ex vivo* Treg cell suppression assays of $CD4^+CD25^+$ Treg cells sorted from tumor-bearing mice showed that Treg cells isolated from KC1-$p53^{KO}$ recipients were more efficient at inhibiting the proliferation of $CD4^+CD25^−$ T cells *in vitro* (Figures 5F and S5F). Both our *in vivo* characterization and *ex vivo* functional assays highlight the relationship between tumor p53 loss and the local and systemic accumulation of regulatory T cell responses.

KRAS Mutations Coordinate with p53 Loss for Myeloid, but Not Treg, Cell Recruitment

In humans, nearly 90% of pancreatic cancer patients show mutations in *KRAS* (cBioportal) (Cerami et al., 2012), and our autochthonous models were based on mutations in KRAS or in upstream signaling receptors (EGFR). Recent work has shown that activating *KRAS* mutations can drive an immunosuppressive response in cancer cells through increased PD-L1 expression (Coelho et al., 2017). In order to examine the role of KRAS activation in promoting tumor tolerance in the context of p53 loss, we used previously described doxycycline (Dox)-inducible $KRAS^{G12D}$-driven p53-null PDAC mouse cells (Ying et al., 2012) (Figure 6A). Tumors were initiated for 7 days in FVB recipient mice treated with Dox to drive mutant KRAS expression before either maintaining Dox treatment ($iKRAS^{G12D}$-ON) or removing Dox ($iKRAS^{G12D}$-OFF). Despite the lack of p53 in all of these tumor cells, removal of Dox resulted in complete eradication of the tumor within 7 days (Figure 6B). We evaluated early and late tumor-associated immune infiltrates at 48 h and 5 days post-Dox withdrawal. Within 48 h of Dox removal, $iKRAS^{G12D}$-OFF tumors displayed reduced frequencies of $CD11b^+$ cells, especially those positive for F4/80 and CXCR3 expression (Figure 6C), with a modest influx of $CD4^+$ T cells but no changes in $CD8^+$ T cell frequencies (Figure 6D). At this time point, $iKRAS^{G12D}$-ON and $iKRAS^{G12D}$-OFF tumors showed no difference in $CD4^+$ T effector cells or $CD4^+FOXP3^+KLRG1^+$ suppressive Treg cells (Figure 6E).

Evaluation of peripheral T cells in recipient mice 5 days after Dox removal showed a significant increase of pro-inflammatory $CD4^+$ and $CD8^+$ T cells in the spleen and a trend toward an increase in the dLNs under $iKRAS^{G12D}$-OFF conditions (Figures S6A and S6B). There was no overall change in suppressive Treg cell populations (Figure S6C). These data suggest that KRAS inactivation in part reactivates T cell effector immune responses but has no impact on the accumulation of regulatory T cells. To further explore the cooperation between p53 loss and KRAS activation, we turned to a previously described model of isogenic C57Bl6/J ID8 ovarian cancer cells with and without p53, which are WT for KRAS (Walton et al., 2016). These cells were injected into the peritoneum of syngeneic C57Bl6/J mice (Figure 6F), and as we previously published, there was a skewing toward PMN cells present in the ascites of mice carrying $p53^{KO}$ tumors (Walton et al., 2016). Interestingly, while there was no change in the total frequency of $CD4^+$ T cells (Figures S6D and S6E), the ascites from mice injected with ID8 $p53^{KO}$ cells showed a 2-fold increase of regulatory T cells (Figures 6G and 6H) and a preferential skewing toward Treg cells, as illustrated by the $FOXP3^+$ to $FOXP3^−$ ratio (Figure S6F). A major site of ovarian cancer spread is to the omental fat or the omentum (Worzfeld et al., 2017). Processing and staining of omental tumors for flow cytometry revealed enriched populations of regulatory T cells present in p53-ablated lesions (Figure 6I). These results further support a model in which p53 loss in tumors promotes Treg cell infiltration.

DISCUSSION

In this study, we showed that p53-deficient tumors can re-orchestrate the innate immune response through suppression of effector $CD4^+$ and $CD8^+$ T cells, which reflected an increase in the inhibitory actions of myeloid suppressor cells and regulatory T cells, accompanied by a direct impairment of GzmB in $CD8^+$ T cells. Other studies have shown that mutations in p53 correlate with reduced GzmB in gastric cancers (Jiang et al., 2018) and a CSF1 (M-CSF) response signature in breast cancers (Beck et al., 2009). Somatic changes in the tumor can also induce systemic changes beyond the tumor microenvironment, and we showed dampened local and systemic T cell effector function in mice harboring p53-deficient tumors, seen as decreased IFN-γ and TNF-α production (Figure 3). We propose that these weakened T cell responses are partially mediated by suppressive myeloid cells derived from the tumor as well as through Treg cell suppressive functions. Conditioned media from p53-null cancer cells alters the cytokine profile of BMDMs, which in turn influences CTL and Th1 cell differentiation (Figure 3). These observations are consistent with previous publications showing that tumor-derived $CD11bGr^+$ cells promote the *de novo* differentiation of Treg cells in prostate cancer models and reduce circulating IFN-γ-producing T cells in B cell malignancies (Bezzi et al., 2018; Christopoulos et al., 2011). Systemic changes were also seen in p53-deficient breast cancer models, which also showed an increase in MCP1 serum levels similar to those seen in our p53-null tumor-bearing mice (Wellenstein et al., 2019).

It is important to note that unlike the response to reexpression of p53 in p53-null cancers, the p53-expressing cells in our study did not undergo senescence and maintained proliferative ability that was similar to the p53-null cells both *in vitro* and *in vivo* (Figure 1). Recent studies have attributed changes in cytokine profiles to oncogenic drivers, such as mutation in KRAS, MYC, and loss of PTEN (Coelho et al., 2017; Kortlever et al., 2017; Peng et al., 2016; Pylayeva-Gupta et al., 2012). Our data suggest that one consequence of p53 loss is enhanced secretion of cytokines involved in myeloid recruitment and proliferation. Our work suggests the loss of p53 in tumors has a complex effect

(G) $CD4^+FOXP3^+$ Treg cells in the ascites. Cohort size n = 4; means are represented as ±SEM.
(H) Representative plot of $CD4^+$ T cells expressing FOXP3 and CD44.
(I) $CD4^+FOXP3^+$ T cells in tumor-immune infiltrates in the omentum Cohort size n = 9–10; means are represented as ±SEM.
A two-way ANOVA was used to analyze *in vivo* growth kinetics, and an unpaired Student's t test was used for all bar graphs. p values are $^*p < 0.05$, $^{**}p < 0.01$, and $^{***}p < 0.001$. See also Figure S6.

on cytokine secretion of both tumor and macrophage populations, ultimately affecting the T cell response. These observations complement recent work showing that an increase in WNT secretion in response to p53 loss in breast cancer cells can stimulate systemic inflammation and drive metastasis (Wellenstein et al., 2019). The complexity of the immune response to p53 loss is further highlighted by a recent study showing that loss of p53 in a prostate cancer model increases expression of CXCL17 and CXCL5 and consequent attraction of tumor-promoting Gr-1$^+$CD11b$^+$ cells (Bezzi et al., 2018). Consistent with our study, loss of p53 in the prostate model also led to an increased expression of CXCL9 and CXCL10 (Bezzi et al., 2018). Although we were unable to identify any changes in the intrinsic ability of p53-null cancer cells to present antigen, previous studies have shown p53-dependent alterations in antigen presentation (Wang et al., 2013; Zhu et al., 1999), and it remains possible that cell-intrinsic changes also play a role in the immune response to p53 loss.

Although blockade of the M-CSF receptor, CSF1R, produced a positive effect in allowing infiltration of T cell effector function in the p53-null tumors, this was not sufficient for rejection. This observation dovetails with a previous study targeting CSF1R with PD-L1 in an orthotopic pancreatic model (with mutations in *KRAS* and loss of *INK*) to increase the efficacy of PD-L1 blockade (Zhu et al., 2014). Blocking CSF1R by chemical targeting in the p53^{R172H} *Kras* pancreatic mouse model has shown a 2-week increase in survival and, as we also showed, an increase in T cell effector function (Candido et al., 2018). In addition, macrophage depletion with clodronate and CSF1R inhibition did not provide therapeutic benefit to primary tumors in the KPC pancreatic cancer model, similar to our results (Griesmann et al., 2017). Given the complexity of the immune modulatory response to loss of p53, it is not surprising that reversal of only one part of the response is insufficient to fully regain tumor rejection, since CD11b$^+$ and Treg cell populations remained enriched in p53-null tumors. By targeting both suppressive populations through double blockade with anti-CSF1-R and anti-CD25, we were able to diminish the growth of p53-null tumors in an MHC-mismatched recipient. Indeed, our data show that the enhanced Treg cell infiltration into p53-null tumors is not prevented by CSF1R blockade, highlighting the importance of a combination immunotherapy.

MSDCs and Treg cells are two major immune-suppressive populations hijacked by many cancer cells. MDSCs arise and expand under pathological conditions, especially during cancer. Naturally occurring Treg cells are a heterogeneous population, with different surface markers predicting suppressive capacity (Cheng et al., 2012). In both our subcutaneous and orthotopic models, we detect an enrichment of KLRG1$^+$ and CLTA4$^+$ Treg cells in p53-null tumor-bearing mice. KLRG1$^+$ Treg cells are considered a highly suppressive and terminally differentiated population of Treg cells that arise during tissue repair, such as influenza-induced lung injury, and help in tissue regeneration (Arpaia et al., 2015). Consistently, Treg cells isolated from recipients harboring p53-deficient tumors displayed increased suppressive capacity compared to their WT counterparts, dovetailing with a similar observation in PTEN- and p53-null prostate tumors (Bezzi et al., 2018).

Pancreatic and lung tumors harboring mutations in *KRAS* also frequently acquire mutations in p53. While loss of p53 permits the proliferation of mutant-KRAS-expressing cells, which would otherwise undergo senescence, our data also demonstrate cooperation between KRAS and p53 mutations in allowing cancer cells to evade the immune response. Previous studies on pancreatic cancer have focused on the effects of mutant KRAS in pancreatic ductal epithelial cells (PDECs) and in combination with mutant p53 in PDAC models, where GM-CSF was a contributing factor for neutrophil recruitment to the tumor (Bayne et al., 2012; Pylayeva-Gupta et al., 2012). Interestingly, while neutrophils were enriched in orthotopically implanted KRAS^{G12D} PDECs, there was no change in Treg cell infiltration (Pylayeva-Gupta et al., 2012), supporting our observation that p53 plays a more important role in modulating Treg cells in cancer. The selection for suppressive Treg cells seems to depend predominantly on p53 loss rather than the coordinated overexpression of mutant KRAS, as demonstrated by the iKRAS system and the ID8 ovarian cancer model. Moreover, a recent study analyzing lung cancer patients treated with PD-L1 blockade reported that patients harboring both *KRAS* and *TP53* mutations were resistant to immunotherapy through a mechanism that was independent of PD-L1 expression levels (Skoulidis et al., 2018). Hence, understanding how different oncogenic drivers interact to promote immune tolerance will be required to understand how best to apply immunomodulatory therapies in cancer.

STAR★METHODS

Detailed methods are provided in the online version of this paper and include the following:

- KEY RESOURCES TABLE
- LEAD CONTACT AND MATERIALS AVAILABILITY
- EXPERIMENTAL MODEL AND SUBJECT DETAILS
  - Mice
  - Cell lines and transfection
  - Mouse Cancer Orthotopic Models
  - Tumor Challenge and Rejection Models
- METHOD DETAILS
  - T cell and CD11b$^+$ cell purification, macrophage differentiation, and cell culture
  - Treg and CD11b suppression assays
  - Flow cytometry
  - Extracellular Cytokine Measurements
  - Immunoblotting
  - *In Vivo* Imaging
  - Co-Culture Assays
  - Tissue collection, Immunohistochemistry (IHC) and scoring
  - Tumor Preparation and Serum Collection
  - RNA extraction and QPCR
  - Scratch-Wound and Migration Assays
  - Plasmids, stable expression and CRISPR/CAS9
  - Bioinformatics Analysis of TCGA datasets
- QUANTIFICATION AND STATISTICAL ANALYSIS
- DATA AND CODE AVAILABILITY

SUPPLEMENTAL INFORMATION

Supplemental Information can be found online at https://doi.org/10.1016/j.celrep.2019.12.028.

ACKNOWLEDGMENTS

We would like to thank the histology team at the Beatson Institute for Cancer Research and the animal facilities at the Francis Crick and Beatson Institutes. J.B. was funded by a Canadian Institute of Health Research (CIHR) postdoctoral grant. J.B.W. was funded by the Marsh Rivkin Center for Ovarian Cancer. S.H. and E.G. were funded by a European Research Council (ERC) THESEUS grant. We would like to acknowledge and thank the Pear lab and Castro lab for donating their plasmids to Addgene (Pear et al., 1998; Yang et al., 2010). This work was funded by Cancer Research UK grants C596/A10419 and C596/A26855, and supported by the Francis Crick Institute, which receives its core funding from Cancer Research UK (FC001557), the UK Medical Research Council (FC001557), and the Wellcome Trust (FC001557), and the CRUK Beatson Institute, which receives its core funding from Cancer Research UK grant C596/A17196. C.S. is Royal Society Napier Research Professor. This work was supported by the Francis Crick Institute, which receives its core funding from Cancer Research UK (FC001169 and FC001202), the UK Medical Research Council (FC001169 and FC001202), and the Wellcome Trust (FC001169 and FC001202). C.S. is funded by Cancer Research UK (TRACERx, PEACE, and CRUK Cancer Immunotherapy Catalyst Network), the CRUK Lung Cancer Centre of Excellence, the Rosetrees Trust, NovoNordisk Foundation (ID16584), and the Breast Cancer Research Foundation (BCRF). This research is supported by a Stand Up To Cancer-LUNGevity-American Lung Association Lung Cancer Interception Dream Team translational research grant (SU2C-AACR-DT23-17). Stand Up To Cancer is a program of the Entertainment Industry Foundation. Research grants are administered by the American Association for Cancer Research, the Scientific Partner of Stand Up To Cancer. C.S. receives funding from the ERC under the European Union's Seventh Framework Programme (FP7/2007-2013) consolidator grant (FP7-THESEUS-617844); European Commission ITN (FP7-PloidyNet 607722); an ERC advanced grant (PROTEUS) from the ERC under the European Union's Horizon 2020 research and innovation program (grant agreement 835297); and Chromavision from the European Union's Horizon 2020 research and innovation program (grant agreement 665233).

AUTHOR CONTRIBUTIONS

The study was conceived and designed by J.B., F.Z., S.H., E.G., M.H., K.B., and K.H.V. Investigations were conducted by J.B., F.Z., M.H., S.P., A.H., S.H., J.B.W., S.M., M.Y., and J.P.M. Bioinformatics analysis was carried out by P.C. The manuscript was written by J.B., F.Z., and K.H.V., and all authors contributed to the review of the paper.

DECLARATION OF INTERESTS

K.H.V. is on the board of directors and shareholder of Bristol Myers Squibb, a shareholder of GRAIL, and on the science advisory board of PMV Pharma, RAZE Therapeutics, and Ludwig Cancer. She is a co-founder and consultant of Faeth Therapeutics, funded by Khosla Ventures. She has been in receipt of research funding from Astex Pharmaceuticals and AstraZeneca and contributed to CRUK Cancer Research Technology filing of patent application WO/2017/144877. C.S. receives grant support from Pfizer, AstraZeneca, BMS, Roche-Ventana, and Boehringer-Ingelheim; has consulted for Pfizer, Novartis, GlaxoSmithKline, MSD, BMS, Celgene, AstraZeneca, Illumina, Genentech, Roche-Ventana, GRAIL, Medicxi, and the Sarah Cannon Research Institute; is an advisor for Dynamo Therapeutics; is a shareholder of Apogen Biotechnologies, Epic Bioscience, and GRAIL; and has stock options in and is co-founder of Achilles Therapeutics. I.A.M. is on the advisory boards for Clovis Oncology, Tesaro, AstraZeneca, Roche, and Carrick Therapeutics and is in receipt of research funding from AstraZeneca.

Received: February 21, 2019
Revised: July 19, 2019
Accepted: December 6, 2019
Published: January 14, 2020

REFERENCES

Ancrile, B.B., O'Hayer, K.M., and Counter, C.M. (2008). Oncogenic ras-induced expression of cytokines: a new target of anti-cancer therapeutics. Mol. Interv. *8*, 22–27.

Arce Vargas, F., Furness, A.J.S., Solomon, I., Joshi, K., Mekkaoui, L., Lesko, M.H., Miranda Rota, E., Dahan, R., Georgiou, A., Sledzinska, A., et al.; Melanoma TRACERx Consortium; Renal TRACERx Consortium; Lung TRACERx Consortium (2017). Fc-optimized anti-CD25 depletes tumor-infiltrating regulatory T cells and synergizes with PD-1 blockade to eradicate established tumors. Immunity *46*, 577–586.

Arpaia, N., Green, J.A., Moltedo, B., Arvey, A., Hemmers, S., Yuan, S., Treuting, P.M., and Rudensky, A.Y. (2015). A distinct function of regulatory T cells in tissue protection. Cell *162*, 1078–1089.

Athie-Morales, V., Smits, H.H., Cantrell, D.A., and Hilkens, C.M. (2004). Sustained IL-12 signaling is required for Th1 development. J. Immunol. *172*, 61–69.

Bankhead, P., Loughrey, M.B., Fernández, J.A., Dombrowski, Y., McArt, D.G., Dunne, P.D., McQuaid, S., Gray, R.T., Murray, L.J., Coleman, H.G., et al. (2017). QuPath: open source software for digital pathology image analysis. Sci. Rep. *7*, 16878.

Bartocci, A., Mastrogiannis, D.S., Migliorati, G., Stockert, R.J., Wolkoff, A.W., and Stanley, E.R. (1987). Macrophages specifically regulate the concentration of their own growth factor in the circulation. Proc. Natl. Acad. Sci. USA *84*, 6179–6183.

Bayne, L.J., Beatty, G.L., Jhala, N., Clark, C.E., Rhim, A.D., Stanger, B.Z., and Vonderheide, R.H. (2012). Tumor-derived granulocyte-macrophage colony-stimulating factor regulates myeloid inflammation and T cell immunity in pancreatic cancer. Cancer Cell *21*, 822–835.

Beck, A.H., Espinosa, I., Edris, B., Li, R., Montgomery, K., Zhu, S., Varma, S., Marinelli, R.J., van de Rijn, M., and West, R.B. (2009). The macrophage colony-stimulating factor 1 response signature in breast carcinoma. Clin. Cancer Res. *15*, 778–787.

Bezzi, M., Seitzer, N., Ishikawa, T., Reschke, M., Chen, M., Wang, G., Mitchell, C., Ng, C., Katon, J., Lunardi, A., et al. (2018). Diverse genetic-driven immune landscapes dictate tumor progression through distinct mechanisms. Nat. Med. *24*, 165–175.

Candido, J.B., Morton, J.P., Bailey, P., Campbell, A.D., Karim, S.A., Jamieson, T., Lapienyte, L., Gopinathan, A., Clark, W., McGhee, E.J., et al. (2018). $CSF1R^+$ macrophages sustain pancreatic tumor growth through T Cell suppression and maintenance of key gene programs that define the squamous subtype. Cell Rep. *23*, 1448–1460.

Cassetta, L., and Pollard, J.W. (2018). Targeting macrophages: therapeutic approaches in cancer. Nat. Rev. Drug Discov. *17*, 887–904.

Cerami, E., Gao, J., Dogrusoz, U., Gross, B.E., Sumer, S.O., Aksoy, B.A., Jacobsen, A., Byrne, C.J., Heuer, M.L., Larsson, E., et al. (2012). The cBio cancer genomics portal: an open platform for exploring multidimensional cancer genomics data. Cancer Discov. *2*, 401–404.

Chang, C.H., Qiu, J., O'Sullivan, D., Buck, M.D., Noguchi, T., Curtis, J.D., Chen, Q., Gindin, M., Gubin, M.M., van der Windt, G.J., et al. (2015). Metabolic competition in the tumor microenvironment is a driver of cancer progression. Cell *162*, 1229–1241.

Cheng, G., Yuan, X., Tsai, M.S., Podack, E.R., Yu, A., and Malek, T.R. (2012). IL-2 receptor signaling is essential for the development of Klrg1+ terminally differentiated T regulatory cells. J. Immunol. *189*, 1780–1791.

Christopoulos, P., Pfeifer, D., Bartholomé, K., Follo, M., Timmer, J., Fisch, P., and Veelken, H. (2011). Definition and characterization of the systemic T-cell dysregulation in untreated indolent B-cell lymphoma and very early CLL. Blood *117*, 3836–3846.

Coelho, M.A., de Carne Trecesson, S., Rana, S., Zecchin, D., Moore, C., Molina-Arcas, M., East, P., Spencer-Dene, B., Nye, E., Barnouin, K., et al. (2017). Oncogenic RAS signaling promotes tumor immunoresistance by stabilizing PD-L1 mRNA. Immunity *47*, 1083–1099.e1086.

Collison, L.W., and Vignali, D.A. (2011). In vitro Treg suppression assays. Methods Mol. Biol. *707*, 21–37.

Cooks, T., Pateras, I.S., Jenkins, L.M., Patel, K.M., Robles, A.I., Morris, J., Forshew, T., Appella, E., Gorgoulis, V.G., and Harris, C.C. (2018). Mutant p53 cancers reprogram macrophages to tumor supporting macrophages via exosomal miR-1246. Nat. Commun. *9*, 771.

Dodge, I.L., Carr, M.W., Cernadas, M., and Brenner, M.B. (2003). IL-6 production by pulmonary dendritic cells impedes Th1 immune responses. J. Immunol. *170*, 4457–4464.

Dunn, G.P., Bruce, A.T., Sheehan, K.C., Shankaran, V., Uppaluri, R., Bui, J.D., Diamond, M.S., Koebel, C.M., Arthur, C., White, J.M., and Schreiber, R.D. (2005). A critical function for type I interferons in cancer immunoediting. Nat. Immunol. *6*, 722–729.

Fischer, K., Hoffmann, P., Voelkl, S., Meidenbauer, N., Ammer, J., Edinger, M., Gottfried, E., Schwarz, S., Rothe, G., Hoves, S., et al. (2007). Inhibitory effect of tumor cell-derived lactic acid on human T cells. Blood *109*, 3812–3819.

George, S., Miao, D., Demetri, G.D., Adeegbe, D., Rodig, S.J., Shukla, S., Lipschitz, M., Amin-Mansour, A., Raut, C.P., Carter, S.L., et al. (2017). Loss of PTEN is associated with resistance to anti-PD-1 checkpoint blockade therapy in metastatic uterine leiomyosarcoma. Immunity *46*, 197–204.

Griesmann, H., Drexel, C., Milosevic, N., Sipos, B., Rosendahl, J., Gress, T.M., and Michl, P. (2017). Pharmacological macrophage inhibition decreases metastasis formation in a genetic model of pancreatic cancer. Gut *66*, 1278–1285.

Guo, G., Marrero, L., Rodriguez, P., Del Valle, L., Ochoa, A., and Cui, Y. (2013). Trp53 inactivation in the tumor microenvironment promotes tumor progression by expanding the immunosuppressive lymphoid-like stromal network. Cancer Res. *73*, 1668–1675.

Guo, G., Yu, M., Xiao, W., Celis, E., and Cui, Y. (2017). Local activation of p53 in the tumor microenvironment overcomes immune suppression and enhances antitumor immunity. Cancer Res. *77*, 2292–2305.

Hock, A.K., Lee, P., Maddocks, O.D., Mason, S.M., Blyth, K., and Vousden, K.H. (2014). iRFP is a sensitive marker for cell number and tumor growth in high-throughput systems. Cell Cycle *13*, 220–226.

Hollstein, M., Sidransky, D., Vogelstein, B., and Harris, C.C. (1991). p53 mutations in human cancers. Science *253*, 49–53.

Iannello, A., Thompson, T.W., Ardolino, M., Lowe, S.W., and Raulet, D.H. (2013). p53-dependent chemokine production by senescent tumor cells supports NKG2D-dependent tumor elimination by natural killer cells. J. Exp. Med. *210*, 2057–2069.

Jiang, Z., Liu, Z., Li, M., Chen, C., and Wang, X. (2018). Immunogenomics analysis reveals that TP53 mutations inhibit tumor immunity in gastric cancer. Transl. Oncol. *11*, 1171–1187.

Jones, R.G., Bui, T., White, C., Madesh, M., Krawczyk, C.M., Lindsten, T., Hawkins, B.J., Kubek, S., Frauwirth, K.A., Wang, Y.L., et al. (2007). The proapoptotic factors Bax and Bak regulate T cell proliferation through control of endoplasmic reticulum Ca(2+) homeostasis. Immunity *27*, 268–280.

Kawashima, H., Takatori, H., Suzuki, K., Iwata, A., Yokota, M., Suto, A., Minamino, T., Hirose, K., and Nakajima, H. (2013). Tumor suppressor p53 inhibits systemic autoimmune diseases by inducing regulatory T cells. J. Immunol. *191*, 3614–3623.

Kim, M.P., Evans, D.B., Wang, H., Abbruzzese, J.L., Fleming, J.B., and Gallick, G.E. (2009). Generation of orthotopic and heterotopic human pancreatic cancer xenografts in immunodeficient mice. Nat. Protoc. *4*, 1670–1680.

Kornete, M., Sgouroudis, E., and Piccirillo, C.A. (2012). ICOS-dependent homeostasis and function of Foxp3+ regulatory T cells in islets of nonobese diabetic mice. J. Immunol. *188*, 1064–1074.

Kortlever, R.M., Sodir, N.M., Wilson, C.H., Burkhart, D.L., Pellegrinet, L., Brown Swigart, L., Littlewood, T.D., and Evan, G.I. (2017). Myc cooperates with Ras by programming inflammation and immune suppression. Cell *171*, 1301–1315.e1314.

Love, M.I., Huber, W., and Anders, S. (2014). Moderated estimation of fold change and dispersion for RNA-seq data with DESeq2. Genome Biol. *15*, 550.

Lowe, J.M., Menendez, D., Bushel, P.R., Shatz, M., Kirk, E.L., Troester, M.A., Garantziotis, S., Fessler, M.B., and Resnick, M.A. (2014). p53 and NF-κB coregulate proinflammatory gene responses in human macrophages. Cancer Res. *74*, 2182–2192.

Lujambio, A., Akkari, L., Simon, J., Grace, D., Tschaharganeh, D.F., Bolden, J.E., Zhao, Z., Thapar, V., Joyce, J.A., Krizhanovsky, V., and Lowe, S.W. (2013). Non-cell-autonomous tumor suppression by p53. Cell *153*, 449–460.

Martinelli, R., Sabroe, I., LaRosa, G., Williams, T.J., and Pease, J.E. (2001). The CC chemokine eotaxin (CCL11) is a partial agonist of CC chemokine receptor 2b. J. Biol. Chem. *276*, 42957–42964.

McGranahan, N., Rosenthal, R., Hiley, C.T., Rowan, A.J., Watkins, T.B.K., Wilson, G.A., Birkbak, N.J., Veeriah, S., Van Loo, P., Herrero, J., et al. (2017). Allele-specific HLA loss and immune escape in lung cancer evolution. Cell *171*, 1259–1271.e1211.

Morton, J.P., Timpson, P., Karim, S.A., Ridgway, R.A., Athineos, D., Doyle, B., Jamieson, N.B., Oien, K.A., Lowy, A.M., Brunton, V.G., et al. (2010). Mutant p53 drives metastasis and overcomes growth arrest/senescence in pancreatic cancer. Proc. Natl. Acad. Sci. USA *107*, 246–251.

Myant, K.B., Cammareri, P., McGhee, E.J., Ridgway, R.A., Huels, D.J., Cordero, J.B., Schwitalla, S., Kalna, G., Ogg, E.L., Athineos, D., et al. (2013). ROS production and NF-κB activation triggered by RAC1 facilitate WNT-driven intestinal stem cell proliferation and colorectal cancer initiation. Cell Stem Cell *12*, 761–773.

Noy, R., and Pollard, J.W. (2014). Tumor-associated macrophages: from mechanisms to therapy. Immunity *41*, 49–61.

Okuda, Y., Okuda, M., and Bernard, C.C. (2003). Regulatory role of p53 in experimental autoimmune encephalomyelitis. J. Neuroimmunol. *135*, 29–37.

Onizuka, S., Tawara, I., Shimizu, J., Sakaguchi, S., Fujita, T., and Nakayama, E. (1999). Tumor rejection by in vivo administration of anti-CD25 (interleukin-2 receptor alpha) monoclonal antibody. Cancer Res. *59*, 3128–3133.

Park, J.S., Lim, M.A., Cho, M.L., Ryu, J.G., Moon, Y.M., Jhun, J.Y., Byun, J.K., Kim, E.K., Hwang, S.Y., Ju, J.H., et al. (2013). p53 controls autoimmune arthritis via STAT-mediated regulation of the Th17 cell/Treg cell balance in mice. Arthritis Rheum. *65*, 949–959.

Pear, W.S., Miller, J.P., Xu, L., Pui, J.C., Soffer, B., Quackenbush, R.C., Pendergast, A.M., Bronson, R., Aster, J.C., Scott, M.L., and Baltimore, D. (1998). Efficient and rapid induction of a chronic myelogenous leukemia-like myeloproliferative disease in mice receiving P210 bcr/abl-transduced bone marrow. Blood *92*, 3780–3792.

Peng, W., Chen, J.Q., Liu, C., Malu, S., Creasy, C., Tetzlaff, M.T., Xu, C., McKenzie, J.A., Zhang, C., Liang, X., et al. (2016). Loss of PTEN promotes resistance to T cell-mediated immunotherapy. Cancer Discov. *6*, 202–216.

Pfaffl, M.W. (2001). A new mathematical model for relative quantification in real-time RT-PCR. Nucleic Acids Res. *29*, e45.

Prenen, H., and Mazzone, M. (2019). Tumor-associated macrophages: a short compendium. Cell. Mol. Life Sci. *76*, 1447–1458.

Pylayeva-Gupta, Y., Lee, K.E., Hajdu, C.H., Miller, G., and Bar-Sagi, D. (2012). Oncogenic Kras-induced GM-CSF production promotes the development of pancreatic neoplasia. Cancer Cell *21*, 836–847.

Qian, B.Z., and Pollard, J.W. (2010). Macrophage diversity enhances tumor progression and metastasis. Cell *141*, 39–51.

Ribas, A., and Wolchok, J.D. (2018). Cancer immunotherapy using checkpoint blockade. Science *359*, 1350–1355.

Sakaguchi, S., Yamaguchi, T., Nomura, T., and Ono, M. (2008). Regulatory T cells and immune tolerance. Cell *133*, 775–787.

Schneider, C.A., Rasband, W.S., and Eliceiri, K.W. (2012). NIH Image to ImageJ: 25 years of image analysis. Nature Methods *9*, 671–675.

Setiady, Y.Y., Coccia, J.A., and Park, P.U. (2010). In vivo depletion of CD4+FOXP3+ Treg cells by the PC61 anti-CD25 monoclonal antibody is mediated by FcgammaRIII+ phagocytes. Eur. J. Immunol. *40*, 780–786.

Shcherbakova, D.M., and Verkhusha, V.V. (2013). Near-infrared fluorescent proteins for multicolor in vivo imaging. Nat. Methods *10*, 751–754.

Shimizu, J., Yamazaki, S., and Sakaguchi, S. (1999). Induction of tumor immunity by removing CD25+CD4+ T cells: a common basis between tumor immunity and autoimmunity. J. Immunol. *163*, 5211–5218.

Siemers, N.O., Holloway, J.L., Chang, H., Chasalow, S.D., Ross-MacDonald, P.B., Voliva, C.F., and Szustakowski, J.D. (2017). Genome-wide association analysis identifies genetic correlates of immune infiltrates in solid tumors. PLoS ONE *12*, e0179726.

Skoulidis, F., Goldberg, M.E., Greenawalt, D.M., Hellmann, M.D., Awad, M.M., Gainor, J.F., Schrock, A.B., Hartmaier, R.J., Trabucco, S.E., Gay, L., et al. (2018). *STK11/LKB1* mutations and PD-1 inhibitor resistance in *KRAS*-mutant lung adenocarcinoma. Cancer Discov. *8*, 822–835.

Spranger, S., Bao, R., and Gajewski, T.F. (2015). Melanoma-intrinsic β-catenin signalling prevents anti-tumour immunity. Nature *523*, 231–235.

Tan, E.H., Morton, J.P., Timpson, P., Tucci, P., Melino, G., Flores, E.R., Sansom, O.J., Vousden, K.H., and Muller, P.A. (2014). Functions of TAp63 and p53 in restraining the development of metastatic cancer. Oncogene *33*, 3325–3333.

Tanaka, A., and Sakaguchi, S. (2017). Regulatory T cells in cancer immunotherapy. Cell Res. *27*, 109–118.

Walton, J., Blagih, J., Ennis, D., Leung, E., Dowson, S., Farquharson, M., Tookman, L.A., Orange, C., Athineos, D., Mason, S., et al. (2016). CRISPR/Cas9-mediated Trp53 and Brca2 knockout to generate improved murine models of ovarian high-grade serous carcinoma. Cancer Res. *76*, 6118–6129.

Wang, B., Niu, D., Lai, L., and Ren, E.C. (2013). p53 increases MHC class I expression by upregulating the endoplasmic reticulum aminopeptidase ERAP1. Nat. Commun. *4*, 2359.

Watanabe, M., Moon, K.D., Vacchio, M.S., Hathcock, K.S., and Hodes, R.J. (2014). Downmodulation of tumor suppressor p53 by T cell receptor signaling is critical for antigen-specific CD4(+) T cell responses. Immunity *40*, 681–691.

Wellenstein, M.D., Coffelt, S.B., Duits, D.E.M., van Miltenburg, M.H., Slagter, M., de Rink, I., Henneman, L., Kas, S.M., Prekovic, S., Hau, C.S., et al. (2019). Loss of p53 triggers WNT-dependent systemic inflammation to drive breast cancer metastasis. Nature *572*, 538–542.

Worzfeld, T., Pogge von Strandmann, E., Huber, M., Adhikary, T., Wagner, U., Reinartz, S., and Müller, R. (2017). The unique molecular and cellular microenvironment of ovarian cancer. Front. Oncol. *7*, 24.

Wu, W., Dietze, K.K., Gibbert, K., Lang, K.S., Trilling, M., Yan, H., Wu, J., Yang, D., Lu, M., Roggendorf, M., et al. (2015). TLR ligand induced IL-6 counter-regulates the anti-viral CD8(+) T cell response during an acute retrovirus infection. Sci. Rep. *5*, 10501.

Xue, W., Zender, L., Miething, C., Dickins, R.A., Hernando, E., Krizhanovsky, V., Cordon-Cardo, C., and Lowe, S.W. (2007). Senescence and tumour clearance is triggered by p53 restoration in murine liver carcinomas. Nature *445*, 656–660.

Yang, J., Sanderson, N.S., Wawrowsky, K., Puntel, M., Castro, M.G., and Lowenstein, P.R. (2010). Kupfer-type immunological synapse characteristics do not predict anti-brain tumor cytolytic T-cell function in vivo. Proc. Natl. Acad. Sci. USA *107*, 4716–4721.

Ying, H., Kimmelman, A.C., Lyssiotis, C.A., Hua, S., Chu, G.C., Fletcher-Sanjikone, E., Locasale, J.W., Son, J., Zhang, H., Coloff, J.L., et al. (2012). Oncogenic Kras maintains pancreatic tumors through regulation of anabolic glucose metabolism. Cell *149*, 656–670.

Yoon, K.W., Byun, S., Kwon, E., Hwang, S.Y., Chu, K., Hiraki, M., Jo, S.H., Weins, A., Hakroush, S., Cebulla, A., et al. (2015). Control of signaling-mediated clearance of apoptotic cells by the tumor suppressor p53. Science *349*, 1261669.

Zhang, S., Zheng, M., Kibe, R., Huang, Y., Marrero, L., Warren, S., Zieske, A.W., Iwakuma, T., Kolls, J.K., and Cui, Y. (2011). Trp53 negatively regulates autoimmunity via the STAT3-Th17 axis. FASEB J. *25*, 2387–2398.

Zheng, S.J., Lamhamedi-Cherradi, S.E., Wang, P., Xu, L., and Chen, Y.H. (2005). Tumor suppressor p53 inhibits autoimmune inflammation and macrophage function. Diabetes *54*, 1423–1428.

Zhu, K., Wang, J., Zhu, J., Jiang, J., Shou, J., and Chen, X. (1999). p53 induces TAP1 and enhances the transport of MHC class I peptides. Oncogene *18*, 7740–7747.

Zhu, Y., Knolhoff, B.L., Meyer, M.A., Nywening, T.M., West, B.L., Luo, J., Wang-Gillam, A., Goedegebuure, S.P., Linehan, D.C., and DeNardo, D.G. (2014). CSF1/CSF1R blockade reprograms tumor-infiltrating macrophages and improves response to T-cell checkpoint immunotherapy in pancreatic cancer models. Cancer Res. *74*, 5057–5069.

STAR★METHODS

KEY RESOURCES TABLE

REAGENT or RESOURCE	SOURCE	IDENTIFIER
Antibodies		
FITC anti-mouse KRLG1	TONBO biosciences	Cat# 35-5893; RRID:AB_2621718
FITC anti-mouse IFN gamma	TONBO Biosciences	Cat# 35-7311-U100; RRID:AB_2621724
FITC anti-mouse CD4	TONBO Biosciences	Cat# 35-0031-U100; RRID:AB_2621665
FITC anti-mouse CD8a	BioLegend	Cat# 100706; RRID:AB_312745
FITC anti-mouse FOXP3	eBioscience	Cat# 11-5773-82; RRID:AB_465243
PE anti-mouse CTLA-4	eBioscience	Cat# 12-1522-82; RRID:AB_465243
PE anti-mouse F4/80	eBioscience	Cat# 12-4801-82; RRID:AB_465923
PE anti-mouse CD4	BioLegend	Cat# 100407; RRID:AB_312692
PE anti-mouse CD8a	eBioscience	Cat# 12-0081-81; RRID:AB_465529
PE anti-mouse FOXP3	TONBO bioscience	Cat# 50-5773-U100; RRID:AB_2621797
PE anti-mouse CD274	eBioscience	Ca# 12-5982-81; RRID:AB_466088
PE anti-human/mouse CD44	eBioscience	Cat# 12-0441-81; RRID:AB_465663
PerCP-Cy5.5 anti-mouse Ki67	BioLegend	Cat# 652424; RRID:AB_2629531
PerCP-Cy5.5 anti-mouse Ly6G	BioLegend	Cat# 127616; RRID:AB_1727563
PeCy7 anti-mouse Granzyme B	eBioscience	Cat# 25-8898-80; RRID:AB_10853338
PeCy7 anti-mouse GITR	BD Pharmigen	Cat# 558140; RRID:AB_647252
PeCy7 anti-mouse CD11b	BD Pharmigen	Cat# 552850; RRID:AB_394491
PeCy7 anti-mouse NKp46	eBioscience	Cat# 25-3351-82; RRID:AB_394491
PeCy7 anti-mouse CD8a	eBioscience	Cat# 25-0081-81; RRID:AB_469583
PeCy7 anti-mouse CD4	TONBO bioscience	Cat# 60-0041-U100; RRID:AB_469583
PeCy7 anti-mouse CD80	eBioscience	Cat# 25-0801-82; RRID:AB_2573370
eFluor450 anti-mouse IL-2	eBioscience	Cat# 48-7021-82; RRID:AB_1944462
vFluor450 anti-mouse CD4	TONBO bioscience	Cat# 75-0042-U100; RRID:AB_2621928
eFluor450 anti-mouse CD8a	eBioscience	Cat# 48-0081-82; RRID:AB_1272198
BV605 anti-mouse Ly6C	BioLegend	Cat# 128036; RRID:AB_2562353
BV605 anti-mouse CXCR3	BioLegend	Cat# 126523; RRID:AB_2561353
BV650 anti-mouse CD8a	eBioscience	Cat# 100555; RRID:AB_2561353
BV650 anti-mouse CD4	BioLegend	Cat# 100555; RRID:AB_2562529
BV650 anti-mouse CD11b	BioLegend	Cat# 101259; RRID:AB_2566568
BV711 anti-mouse CD11b	BioLegend	Cat# 101242; RRID:AB_2563310
BV711 anti-mouse CD4	BioLegend	Cat# 100557; RRID:AB_2562607
BV711 anti-mouse CD8a	BioLegend	Cat# 100759; RRID:AB_2563510
BV785 anti-mouse MHCII I-A/I-E	BioLegend	Cat# 107646; RRID:AB_313317
BV785 anti-mouse B220	BioLegend	Cat# 103246; RRID:AB_2563256
APC anti-mouse CD4	eBioscience	Cat# 17-0042-82; RRID:AB_469323
APC anti-mouse FOXP3	eBioscience	Cat# 20-0191-U100; RRID:AB_2621561
APC anti-mouse MHCI H-2Kd,H-2Dd	eBioscience	Cat# 17-5998-82; RRID:AB_2573250
APC anti-mouse TNF alpha	eBioscience	Cat# 506308; RRID:AB_315429
APC anti-mouse CD25	eBioscience	Cat# 20-0251-U100; RRID:AB_2621567
APC anti-mouse CD86	eBioscience	Cat# 17-0862-81; RRID:AB_469418
AlexaFluor647 anti-mouse CCR2 (CD192)	BioLegend	Cat# 150604; RRID:AB_2566140
Vinculin mouse monoclonal	Santa Cruz Biotechnology	Cat# SC-73614; RRID:AB_1131294
p53 mouse monoclonal antibody	Cell Signaling Technology	Cat# 2524; RRID:AB_331743

(*Continued on next page*)

Continued

REAGENT or RESOURCE	SOURCE	IDENTIFIER
InVivoPlus mouse IgG2a isotype control	BioXCell	Cat# BP0085; RRID:AB_1107771
InVivo monoclonal anti mouse CSF1R	BioXCell	Cat#BE0213; RRID:AB_466565
InVivo monoclonal anti-CD25	BioXCell	Cat# BE0012; RRID:AB_1107619
Anti-mouse CD28 Functional grade purified	eBioscience	Cat# 16-0281-86; RRID:AB_468923
Anti-mouse CD3e Functional grade purified	eBioscience	Cat# 16-0031-86; RRID:AB_468849
IRDye® 800CW Donkey anti-mouse	Li-Cor	Cat# 926-32212; RRID:AB_621847
IRDye® 680LT Donkey anti-Rabbit	Li-Cor	Cat# 926-68023; RRID:AB_10706167
Chemicals, Peptides, and Recombinant Proteins		
Recombinant Murine Il-2	Peprotech	Cat# 212-12
Ovalbumin peptide 257-364	Francis Crick Institute	N/A
Ovalbumin peptide 323-339	Francis Crick Institute	N/A
Recombinant Murine M-CSF	Peprotech	Cat# 315-02
Critical Commercial Assays		
Mouse M-CSF ELISA Kit	RayBio ®	Cat# ELM-MCSF
Mouse G-CSF ELISA Kit	RayBio ®	Cat# ELM-GCSF
Mouse CXCL1/KC ELISA Kit	OriGene Techologies, Inc	Cat# EA100460
Mouse Cytokine Array Panel A - Proteome Profiler	R&D systems ®	Cat# ARY006
Mouse CCL2 Uncoated ELISA Kit	Invitrogen	Cat# 99-7391-22
Mouse MIP1 alpha (CCL3) Uncoated ELISA Kit	Theromo Fisher Scientific	Cat# 88-56013-22
Mouse LIX ELISA Kit	RayBio ®	Cat# ELM-LIX
ProcartaPlex Mouse Cytokine & Chemokine Panel 1 (26 plex)	Invitrogen eBioscience	EPX260-26088-901
LEGEND MAX Mouse CCL11 ELISA Kit	BioLegend	Cat# 4438907
High capacity cDNA reversts transcription kit	AppliedBiosystems	Cat# 4368814
RNeasy ® Mini Kit	QIAGEN	Cat# 157029548
RNase Free and DNase set	QIAGEN	Cat# 79254
Deposited Data		
PAAD - TCGA	TCGA	https://gdac.broadinstitute.org/; RRID:SCR_003193
Experimental Models: Cell Lines		
Phoenix-ECO	ATCC®	Cat# CRL-3214; RRID:CVCL_H717
KC1 *Pdx1-cre; LSL-KRas*^{G12D}	Beatson Institute for Cancer Research	N/A
KC3 *Pdx1-cre; LSL-KRas*^{G12D}	Beatson Institute for Cancer Research	N/A
KC3 *Pdx1-cre; LSL-KRas*^{G12D}	Beatson Institute for Cancer Research	N/A
KFC1 *Pdx1-cre; LSL-Kras*^{G12D}; *Trp53*$^{fl/+}$	Beatson Institute for Cancer Research	N/A
KFC2 *Pdx1-cre; LSL-Kras*^{G12D}; *Trp53*$^{fl/+}$	Beatson Institute for Cancer Research	N/A
KFC3 *Pdx1-cre; LSL-Kras*^{G12D}; *Trp53*$^{fl/+}$	Beatson Institute for Cancer Research	N/A
ID8	Walton et al., 2016	N/A
ID8-p53^{KO}	Walton et al., 2016	N/A
iKRAS;p53$^{f/+}$	Ying et al., 2012	N/A
Experimental Models: Organisms/Strains		
C57Bl6/J mice females (6-10weeks)	Charles River Laboratories	CR: 632
CD1 nude- Crl:CD1-*Foxn1*$^{nu./nu.}$ females (6-10weeks)	Charles River Laboratories	CR: 087
FVB/NCrl females (6-10weeks)	Charles River Laboratories	CR: 207
Oligonucleotides		
Primers for qPCR T cell cytokines See Table 1	This paper	N/A
Trp53 gRNA/Cas9	Walton et al., 2016	N/A

(*Continued on next page*)

Continued

REAGENT or RESOURCE	SOURCE	IDENTIFIER
Recombinant DNA		
pBABE-iRFP plasmid	Hock et al., 2014	N/A
pCI-neo-mOVA	Yang et al., 2010	Addgene Cat# 25099; RRID:Addgene_25099
MIGR1	Pear et al., 1998	Addgene Cat# 27490; RRID:Addgene_27490
Software and Algorithms		
ImageJ	Schneider et al., 2012	https://imagej.nih.gov/ij/; RRID:SCR_001935
Image Studio v5	Li-Cor	N/A
FlowJo version 9.0	FlowJo	https://www.flowjo.com/solutions/flowjo/downloads; RRID:SCR_008520
survival package version 2.41-3		https://cran.r-project.org/web/packages/surviva/index.html
R version 3.4.3	RStudio	http://www.R-project.org; RRID:SCR_000432
Prism Version 7	GraphPad	https://www.graphpad.com/scientific-software/prism/; RRID:SCR_002798

LEAD CONTACT AND MATERIALS AVAILABILITY

Further information and requests for resources and reagents should be directed to and will be fulfilled by the Lead Contact Karen H Vousden (karen.vousden@crick.ac.uk). All unique/stable reagents generated in this study are available from the Lead Contact with a completed MTA.

EXPERIMENTAL MODEL AND SUBJECT DETAILS

Mice

The mouse models of pancreatic ductal adenocarcinomas were generated using *Pdx1-Cre; LSL-Kras*$^{G12D/+}$*; Trp53*$^{+/+}$ and *Pdx1-Cre; LSL-Kras*$^{G12D/+}$*; Trp53*$^{fl/+}$ as previously published (Morton et al., 2010). The mouse model of non-small cell lung carcinomas was based on the tetracycline inducible EGFR-L858R [Tg(tet-O-EGFR*L858R)56Hev] from the Mouse Repository of the National Cancer Institute. The R26tTA and *Trp53*$^{fl/fl}$ mice were obtained from the Jackson laboratory. Mice were crossed to generate Rosa26tTa^{LSL} tet(O)EGFR^{L858R} and Rosa26tTa^{LSL} tet(O)EGFR^{L858R} *Trp53*$^{flox/flox}$ mice and all backcrossed to C57Bl6/J background. Adenoviral Cre (Viral Vector Core, University of Iowa, USA) was delivered via intratracheal intubation (single dose, 2.5x10^{7} virus particles in 50 μl). FVB (male and females) and CD1$^{nu/nu}$ (females) were purchased from Charles River, and OTI and OTII mice were purchased from Jax and maintained at the Beatson Institute for Cancer Research and the Francis Crick Institute animal facilities. All animals used ranged from 10-30 weeks of age and littermates of the same sex were randomly assigned to experimental groups.

Animal experiments were subject to ethical review by the Francis Crick Animal Welfare and Ethical Review Body and regulation by the UK Home Office project license P8AA77917 and P319AE968 or at the BICR reviewed and approved by the University of Glasgow and UK Home Office for the project license (70/8645). All mice were housed under conditions in line with the Home Office guidelines (UK). Mice were housed from 3-5 per cage and were kept in a 12-hour day/night cycle starting at 7:00 until 19:00. Food and water were available *ad libitum* and rooms were kept at 21°C at 55% humidity. All procedures were performed following the Animals (scientific procedures) Act 1986 and the EU Directive 2010.

Cell lines and transfection

Phoenix-ECO (ATCC® CRL-3214™) were purchased from the ATCC. PDAC cells from *Pdx1-Cre; LSL-Kras*$^{G12D/+}$*; Trp53*$^{+/+}$ and *Pdx1-Cre; LSL-Kras*$^{G12D/+}$*; Trp53*$^{fl/+}$ mice were derived as previously described (Tan et al., 2014). Cell lines used (KCs and KFCs) were tested for genetic background purity by the Charles River Genetic Testing Services. After a 384 SNP panel batch analysis (MB-160318AJ), cell lines derived from tumors were deemed mismatched (e.g., B6N 73%–78.9%, FVB 46.7%–59.8%, 129S4SvJae 60.3%–71.7%). ID8 isogenic ovarian cancer cells were previously described (Walton et al., 2016). iKRAS cell line was gifted by RA DePinho and were maintained in DMEM, 10% FBS and 2μg/ml of doxycycline (Ying et al., 2012). All other cell lines were maintained in DMEM, 10% FBS and penicillin-streptomycin and in 37°C, 5% CO_2 humidified incubators.

Primary T cells and bone marrow were derived from C57Bl6/J mice (either male or female) held at the BICR or Francis Crick institute and aged between 10-30 weeks.

Mouse Cancer Orthotopic Models

The pancreatic orthotopic model protocol was described by Kim et al. (2009). Briefly, pancreatic cancer cells were surgically implanted into the pancreas of recipient mice (500,000 cells in 25μl of Matrigel, BD-Biosciences). For the ovarian ID8 cancer model, $5x10^6$ cells were injected intraperitoneally (IP) in 6-8-week-old female C57Bl6/J mice (Charles River Laboratories, UK). The development of ascites and other symptoms were diagnosed as previously described (Walton et al., 2016).

Tumor Challenge and Rejection Models

PDAC derived cells expressing iRFP were subcutaneously injected into the left flank of FVB mice at $1x10^6$ cells/mouse. Growth was monitored by *in vivo* imaging and mice were taken at humane endpoint as dictated by the UK Home Office and the animal license. Allograft growth in $CD1^{nu/nu}$ mice was performed by unilateral flank injections of $1x10^6$ per mouse. Growth was measured by *in vivo* imaging and humane endpoints were respected. All *in vivo* antibodies were purchased from Bio-X-Cell. Anti-CSF1R (clone AFS98) was used at a concentration of 300μg/mouse and administered twice a week. Anti-CD25 (PC-61.5.3) was used three days prior to tumor challenge and used at 400μg/mouse. Isotype control used was IgG2a (C1.18.4) and 200μg/mouse was used as a control.

METHOD DETAILS

T cell and $CD11b^+$ cell purification, macrophage differentiation, and cell culture

$CD8^+$ T cells and $CD4^+$ T cells were isolated from spleens and peripheral lymph nodes, prepared into single cell suspensions and lysed for red blood cells (10x RBC lysis buffer, Biolegend). Negative isolation kits and positive isolation kits ($CD11b^+$ cells) were purchased from StemCell Technologies and isolation was performed following manufacture's procedures. T cells were activated and cultured as previously described (Jones et al., 2007) using plate-bound anti-CD3 and anti-CD28 antibodies.

Isolation of intratumoral $CD11b^+$ cells was performed on tumors digested using the tumor preparation protocol (see below). $CD11b^+$ cells were isolated from tumor single cell suspensions using positive isolation kits from StemCell Technologies, following the manufacturer's protocol.

Macrophages were differentiated from bone marrow flushed using PBS, a 1mL syringe and a 25G needle. Bone marrow was collected, lysed for red blood cells (10x RBC lysis buffer, Biolegend), and plated on non-TC treated plates at $5x10^6$ cells/10cm dish and 20ng/ml of M-CSF (Peprotech).

Cell culture of pancreatic ductal adenocarcinoma cells (PDACs) and ID8 cell lines were maintained in DMEM supplemented with 10% FBS and Pen/Strep.

Treg and CD11b suppression assays

$CD4^+CD25^+$ and $CD4^+CD25^-$ T cells were isolated by performing $CD4^+$ T cell negative isolation by STEMCELL technology kits and sorted by flow cytometry (FACS Aria Sorter) (CD4-FITC and CD25-APC). Briefly, ratios of 1:1 (Treg:Teffector) to 1:32 were generated in the presence of irradiated splenocytes and activated with 1μg/ml of αCD3 for 3 days (Collison and Vignali, 2011).

$CD11b^+$ cells were isolated by positive selection from digested tumors (see above). T cells were stained with V450 dye and plate-bound activated with a-CD3 (5μg/ml) and a-CD28 (2μg/ml) for 24 hours. After 24 hours, T cells were co-cultured with isolated tumor-associated $CD11b^+$ cells at a ratio of 1:4 in a 24 well plate for a further 2 days and proliferation was assessed using a the eBioscience live/dead fixable viability dye, APC-Cy7, and flow cytometry. Isolated $CD11b^+$ intratumoral cells were plated 8 hours prior to co-culture at $0.5x10^6$ cells per 24 well plate (Corning). Plate-bound activated T cells were plated 24 hours post-activation at $0.75x10^6$ cells per 24 well plate.

Flow cytometry

Single cell suspensions were stained for surface markers in PBS for 20 minutes at 4°C. Intracellular proteins (i.e., cytokines, FOXP3, and Ki67) were assessed using the FOXP3/Transcription staining buffer set (eBioscience, San Diego, CA) and following manufacturer's instructions. Cells were permeabilized for 30 minutes and stained for intracellular proteins for 1 hour at 4°C. All fluorochromes were purchased from Biolegened and eBiosciences. *Ex vivo* re-stimulation was performed using PMA (Sigma-Aldrich), ionomycin (Sigma-Aldrich), and Golgi Stop (BD Biosciences) for 4 hours as previously described (Kornete et al., 2012). For OTI and OTII peptide re-stimulation, peptides OVA257-264 and OVA323-339, were incubated with transgenic T cells at 10μg/ml with Golgi Stop (BD Biosciences) for 6 hours followed by surface staining and ICS. Dead cells were distinguished using the fixable viability dye efluor780® from eBioscience. Single cell suspensions were fixed and permeabilized using the FOXP3 Transcription staining buffer set. Samples were acquired on the BD LSRFortessa™ and on the BD FACSymphony™. Flow cytometry data was analyzed using FlowJo (TreeStar).

Extracellular Cytokine Measurements

ELISA kits were purchased from R&D systems for M-CSF, CXCL1, CCL11, and G-CSF. ELISAs for MCP1, CCL3 and CXCL5 were purchased from Life Technologies, Invitrogen. All ELISAs and cytokine arrays were performed on conditioned media (cells were plated at $1x10^6$ cells/10cm dish) harvested after 24 hours. Cytokine arrays were purchased from R&D systems ® (Mouse cytokine panel array A) and performed on conditioned media from cells prepared as for ELISAs. Cytokines were detected following the

manufacture's procedures and using chemiluminescence (read at 450nm). Pixels from cytokine array data were analyzed using ImageJ software. The Luminex cytokine array used in this study was the Invitrogen eBioscience ProcartaPlex Mouse Cytokine & Chemokine Panel 1 (26 plex) with 4 additional cytokines

Immunoblotting

Cells were lysed using RIPA buffer supplemented the 1%SDS and phosphatase inhibitors (La Roche Ltd), denatured at 95°C, and resolved on NuPAGE polyacrylamide pre-cast gels (ThermoFischer Scientific). Transfer of gels onto nitrocellulose membranes was performed using the iBlot2 (Invitrogen). Cells were probed for p53 with monoclonal anti-mouse p53 antibody from Cell Signaling Technologies (clone 1C12). Vinculin (H10, Santa Cruz Biotechnologies) was detected as a loading control. Secondary antibodies were purchased from LiCOR IRDye 800CW and 700CW.

In Vivo Imaging

Mice were anesthetized with isoflurane and imaged using the Pearl Imager by LiCOR. iRFP fluorescence was excited using the 685nm laser and emission was detected in the 700nm channel (730nm). Fluorescence was analyzed using the Image Studio v5 from LiCOR.

Co-Culture Assays

Activated T cells and PDACS

Primary T cells were activated with plate-bound anti-CD3 (5μg/ml) and anti-CD28 (2μg/ml) for 24 hours. Activated T cells were then co-cultured with adherent PDAC cells in 24 well plates or 96 well plates. PDACs were plated at 20 000 cells/well with $1x10^6$ T cells (24 well plate) or 10,000 PDAC cells/well with 200,000 T cells (96 well plate) and co-incubated for 2 days. T cells were re-stimulated with PMA, ionomycin and GolgiStop (BD Biosciences) for 4 hours prior to ICS. Intracellular cytokines probed by ICS and flow cytometry were Granzyme B, IFNγ and TNFα.

BMDMs and Transgenic T cells

$0.3x10^6$ BMDMs pulsed with 10μg/ml of ovalbumin (Sigma-Aldrich) for 1 hour, washed and naive OTI or OTII cells were added to the culture at $1x10^6$ cells/well. T cells were kept in co-culture for 3-4 days followed by OVA257-264 and OVA323-339 re-stimulation at 10μg/ml in the presence of a Golgi blocker (GolgiStop, BD Bioscience), surface staining, ICS and acquired by flow cytometry.

OTI and PDAC-mOVA cell viability

Splenocytes from OT1 TCR transgenic mice were stimulated *in vitro* with 10μg/ml of SIINFEKL (OVA257-264) for 48 hours. Cells were spun down using Lympholyte M (Cedarlane) following the manufacturer's instructions. Activated CD8+OT1 cells were co-cultured in a 24 well plate (Corning) at $0.5x10^6$ cells to 200,000 PDAC-mOVA isogenic cells. Cells were stained with propidium iodide for cell viability and acquired on the BDLSRFortessa™.

Tissue collection, Immunohistochemistry (IHC) and scoring

Tissue was collected and fixed in 10% neutral buffered formalin (NBF). Samples were replaced with 70% ethanol after 48hours, embedded in paraffin blocks, and processed by standard histological techniques. Sections were cut at 5μM. IHC and H&E was performed as previously published (Myant et al., 2013). F480 antibody was purchased from eBioscience (rat anti-mouse F4/80 Cat. No 14-4801-82; RRID:AB_467558). Scoring of tumor sections for individual markers was performed by counting 30 fields using QuPath open source digital software (Bankhead et al., 2017) and set as an average per field.

Tumor Preparation and Serum Collection

Tumors

Tumors were carefully excised from the animals and kept in ice cold medium till processing. Tumors were minced into small (about 1mm) pieces and digested in digestion buffer containing: collagenase 0.012%, dispase, 0.1mg/ml DNase I, 1% FBS in Krebs Ringer Bicarbonate Buffer (KRB) for 45-60 min at 37°C with gentle oscillation. The digestion was stopped by the addition of at least 10 volume of ice cold DMEM supplemented with 10% FBS. The solution was filtered through a 100 μm cell strainer and the isolated cells were precipitated by centrifugation at 300 X g for 5 min, washed with PBS before further processing.

Serum

Serum was collected by cardiac puncture into EDTA coated tubes. Blood was spun in 1.5mL Eppendorf tubes at 2000xg for 15 minutes at 4°C. Serum was collected in upper phase and stored at −80°C.

RNA extraction and QPCR

Total RNA was extracted using RNeasy® columns (QIAGEN) from at least 3 technical replicates per sample according to manufacturer instructions. Genomic DNA was removed using on column DNA digestion (QIAGEN). cDNA was generated using the High-Capacity cDNA reverse transcription kit (Thermofisher) according to manufacturer's instructions. Power up ™ SYBR® Green Master MIX (Applied Biosystem) was used to perform QPCR with the following primers:

Table 1

Gene and accession number	Forward primer	Reverse primer
Il-6	tcctaccccaatttccaatgctc	ttggatggtcttggtccttagcc
Tnf-α	CCCCAAAGGGATGAGAAGTT	CTCCTCCACTTGGTGGTTTG
IL-12b	CTGCTGCTCCACAAGAAGGA	ACGCCATTCCACATGTCACT
Il-1β	GCAACTGTTCCTGAACTCAACT	TCTTTTGGGGTCCGTCAACT
cyc	ATGGTCAACCCCACCGTGT	TTTCTGCTGTCTTTGGAACTTTGTC

Gene expression values were calculated according to Pfaffl method (Pfaffl, 2001) and expressed as relative units compared to the control group

Scratch-Wound and Migration Assays

Scratch-wound Assay

BMDMs were differentiated with 10ng/ml M-CSF and re-plated onto Imagelock 96 well plates. Scratches were made using the Essenbio scratch making tool. Wound closure was monitored in real-time using IncuCyte™ Live-Cell Analysis System and analyzed using IncuCyte S3 software.

Chemoattraction assays

BMDMs at 5000 cells per chamber were plated in the upper chamber of an IncuCyte™ ClearView 96-well Cell Migration plate (Essen BioScience). Conditioned medium from different PDAC cell lines was placed in the bottom chamber. The Incucyte ZOOM® live-cell imaging system was used to measure cell migration.

Plasmids, stable expression and CRISPR/CAS9

Plasmids for iRFP (Hock et al., 2014) were transfected into Phoenix-ECO (ATCC® CRL-3214) using GeneJuice® Transfection reagents (Merk Milipore). PDAC cell lines were transduced with viral media and pBABE-iRFP selected for by puromycin (Sigma-Aldrich) at a concentration of 2.5μg/ml. Plasmid positive cells were cultured in selection media for 7 days. CRISPR/CAS9 methods were employed for genetic deletion of the *Trp53* gene as previously described (Walton et al., 2016). Retroviral introduction of ovalbumin sequence for membrane bound ovalbumin (mOVA) was cloned out of the pCI-neo-mOVA plasmid (Addgene No 25099) (Yang et al., 2010) and into the MSCV-based MIGR1 (Addgene 27490) vector (Pear et al., 1998). Phoenix-EC (ATCC ® CRL-3214) were used for virus production and PDAC cells were virally transduced three consecutive days. mOVA expressing cells were selected by cell sorting for GFP expression.

Bioinformatics Analysis of TCGA datasets

Survival curves & gene expression: SEM normalized data was downloaded from the TCGA firehose website [https://gdac.broadinstitute.org/]. Top (high group) and bottom (low group) quartiles pancreatic adenocarcinoma samples for MDSC-signature ranked expression were compared using the survival package version 2.41-3 (https://cran.r-project.org/web/packages/survival/index.html) in R version 3.4.3 (http://www.R-project.org) and the level of statistical significance determined by the log rank test. Expression data from PAAD patients with a mutation in *TP53* was compared to those without a mutation using DESEQ2 (Love et al., 2014). Boxplots show the log2 expression values of selected genes grouped by their *TP53* mutation status, with the adjusted p value determined by the Wald test.

QUANTIFICATION AND STATISTICAL ANALYSIS

Data are presented as mean ± SD for technical replicates, or mean ± SEM for biological replicates. Data was analyzed using the unpaired Student t test when comparing two conditions. One-way ANOVA with a Tukey's multiple comparisons test was performed on comparisons of more than two conditions as well as *in vivo* growth studies. Two-way Anova was performed on the *in vivo* tumor growth studies with treatments and different p53 alterations. Statistical significance is indicated in all figures by the following annotations: * $p < 0.05$; ** $p < 0.01$; *** $p < 0.005$. GraphPad Prism 7 was used for statistical analysis and graph generation.

DATA AND CODE AVAILABILITY

This study did not generate any unique datasets or code

Cell Reports, Volume *30*

Supplemental Information

Cancer-Specific Loss of p53 Leads to a Modulation of Myeloid and T Cell Responses

Julianna Blagih, Fabio Zani, Probir Chakravarty, Marc Hennequart, Steven Pilley, Sebastijan Hobor, Andreas K. Hock, Josephine B. Walton, Jennifer P. Morton, Eva Gronroos, Susan Mason, Ming Yang, Iain McNeish, Charles Swanton, Karen Blyth, and Karen H. Vousden

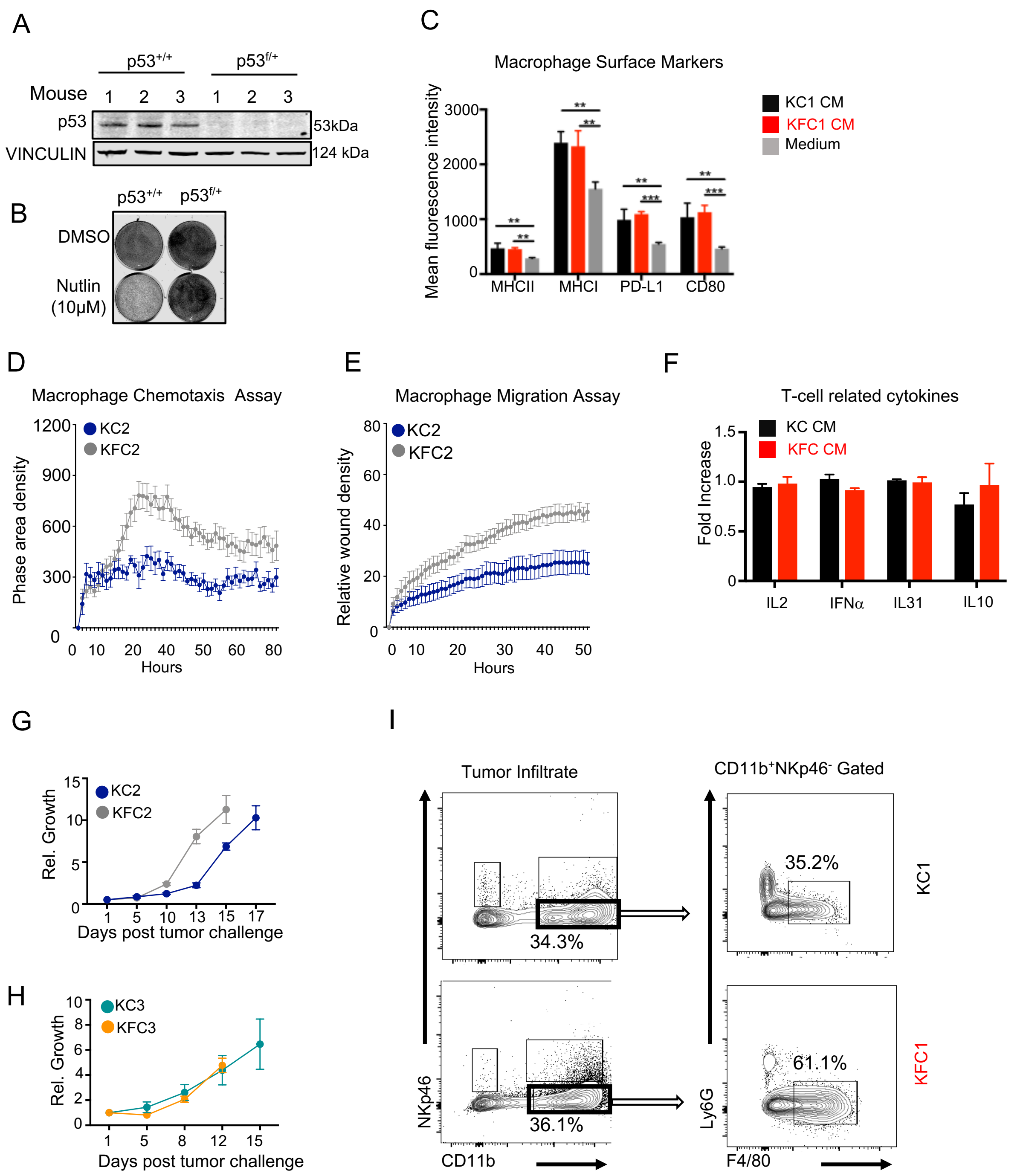

Figure S1. Loss of p53 promotes macrophage migration and infiltration in athymic recipients, Related to Figure 1

A. Immunoblot analysis of *Pdx1-cre; LSL-Kras*$^{G12D/+}$ (KC) and *Pdx1-cre; LSL-Kras*$^{G12D/+}$*; Trp53*$^{fl/+}$ (KFC) tumor-derived cell lines for p53 protein expression. VINCULIN was detected as a loading control. **B.** crystal violet assay of a *Trp53*$^{+/+}$ PDAC derived cell line and a *Trp53*$^{f/+}$ derived cell line in the presence of 10µM Nutlin for 48 hours. **C.** Bone marrow derived macrophages (BMDMs) were activated with medium (grey) or conditioned medium from the KC1 (black) or KFC1 (red) cell lines for 36 hours. Surface expression of MHCII, MHCI, PD-L1, and CD80 was assessed by flow cytometry. Graph represents one of three individual experiments and the mean ±SD of technical replicates (n=5). **D-E.**

Incucyte data from macrophage chemotaxis/migration assays with the mean represented as ±SD. (D) BMDM chemotaxis assay from conditioned medium from another set of PDAC-derived cell lines - the KC2 (blue) and KFC2 (grey) tumor-derived cell lines - measured over 80 hours. (E) BMDM migration-scratch wound assays looking at wound closure in the presence of conditioned medium from the KC2 (blue) and KFC2 (grey) cell lines over 50 hours measuring relative wound density. **F.** Luminex cytokine array performed on conditioned media from 3 independently derived KC (black) and KFC (red) cell lines. IL2, IFNα, IL31, and IL10 were detected. Graph shows fold change from one KC cell line and the mean is represented as ±SEM of biological replicates. **G-H.** Subcutaneous growth of 2 independent p53 WT cell lines, KC2 (blue) and KC3 (teal), and p53-null cell lines, KFC2 (grey) and KFC3 (orange) in CD1$^{nu/nu}$ recipients with the mean represented as ±SEM of cohort sizes of n=5. All tumor derived cell lines were transduced with iRFP and growth was measured as relative growth to fluorescence at day 1 post injection by the Pearl Imager. **I.** Flow cytometry representative plot of intratumoral surface expression of Nkp46$^+$ and CD11b$^+$ immune cells (left) from individual tumors arising either from the KC1 or KFC1 cell line. Right representative plots are gated on CD11b$^+$Nkp46$^-$ populations and analyzed for F4/80 and Ly6G expression of tumors derived from the KC1 and KFC1 cell lines. Statistical p-values are denoted as *p<0.05 and ** p< p<0.01.

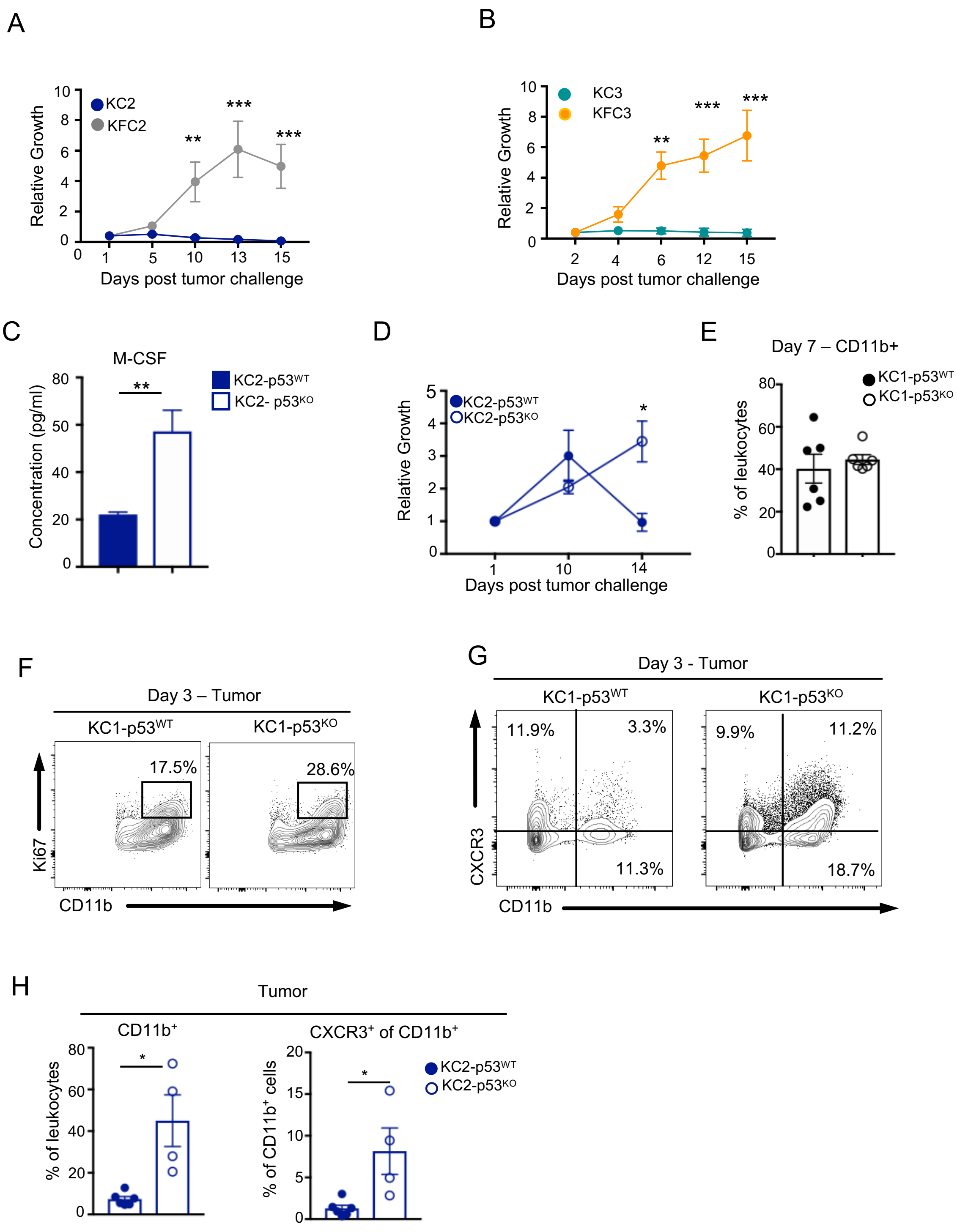

Figure S2. p53 deletion retards tumor regression and increases myeloid infiltration, Related to Figure 2

A-B. Real-time monitoring of tumor growth in MHC-mismatched hosts (FVB strain) by *in vivo* imaging using the Pearl imager with cohort sizes of n=5. Relative subcutaneous growth of a two independent sets of PDAC cell lines of both genotypes - (A) KC2 (blue) and KFC2 (grey) and (B) KC3 (teal) and KFC3 (orange) - transduced with iRFP. Relative growth is represented as the change in infrared fluorescence from day 1 post injection and the mean as ±SEM. **C.** Enzyme-linked immunosorbent assay (ELISA) for M-CSF in conditioned media from the isogenic cell lines, KC2- p53^{WT} (blue) and CRISPR knockout, KC2-p53^{KO} (open). The mean is represented as ±SD and the graph represents one of three independent

experiments. **D.** Monitoring tumor growth over 14 days by *in vivo* imaging of subcutaneous growth of isogenic cell lines – KC2-p53^{WT} and CRISPR knockout for *Trp53*, KC2-p53^{KO}, in FVB MHC-mismatched recipients. Donor cells were transduced with iRFP and growth was made relative to infrared fluorescence day 1 post injection. Cohort sizes were n=5 and the mean measured as ±SEM. One-way ANOVA was used for statistical analysis. **E-G.** Flow cytometry analysis of individual tumor digests of the KC1-p53^{WT} and KC1-p53^{KO} cell lines injected into FVB recipients with cohort sizes of n=6-7 and the mean represented as ±SEM. (E) surface expression for CD11b^{+} in tumor-immune infiltrates of KC1-p53^{WT} and KC1-p53^{KO} tumors at day 7 post injection. (F) Representative flow cytometry plot for tumor immune infiltrates expressing CD11b (X-axis) and Ki67 (Y-axis) of tumor digests at day 3 post injection. (G) Flow cytometry representative plots of KC1-p53^{WT} and KC1-p53^{KO} tumor immune infiltrates at day 3 looking at CD11b (x-axis) CXCR3 (y-axis) expression. **H.** Graphs show frequencies of CD11b^{+} infiltrates and CD11b^{+}CXCR3^{+} immune infiltrates at day 3 post tumor challenge in FVB mice (n=4-5) injected with the KC2-p53^{WT} and KC2-p53^{KO} cell lines with the mean represented as ±SEM. An unpaired Student's t-test was performed unless otherwise stated. Statistical p-values are *p<0.05 and ** p<0.01.

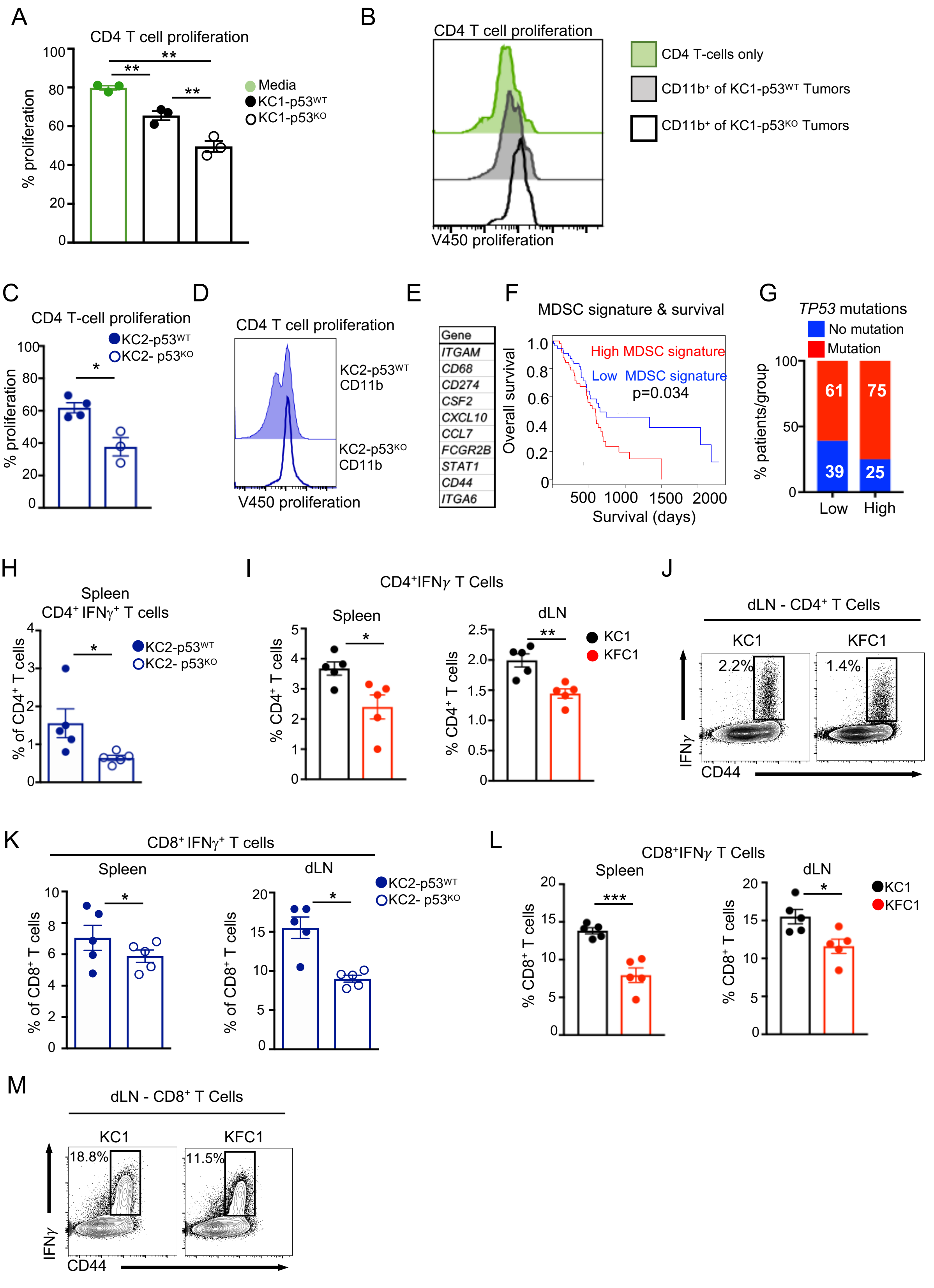

Figure S3. Tumors null for p53 repurpose myeloid cells and dampen T-cell response, Related to Figure 3

A-D. Tumor-associated CD11b$^+$ T cell suppression assays. Graph (A) and flow cytometry plots (B) represent CD4$^+$ T-cell proliferation either alone (green) or co-cultured with CD11b$^+$ cells isolated from KC1-p53^{WT} (grey) or KC1-p53^{KO} (white) tumors. Tukey's multiple comparison test was applied for statistical analysis and the mean is represented as ±SEM with each dot representing one CD11b$^+$ population derived from one individual tumor (n=3). (C) CD11b suppression assay with tumor-derived CD11b$^+$ cells from KC2-p53^{WT} tumors (blue) and KC2-p53^{KO} tumors (open circle) co-cultured with *in vitro* activated CD4$^+$ T cells (n=4-5). The mean is represented as ±SEM and an unpaired Student's t test was applied. (D) Representative T-cell proliferation blot of v450 proliferation dye dilution after 48 hours of co-incubation. **E-G.** Analysis of the pancreatic cancer patient cohort (PAAD) from The Cancer Genome Atlas (TCGA). (E) Myeloid derived suppressor cell (MDSC) gene signature used to define MDSC high (red) and low (blue) subsets in PAAD TCGA cohort. Sample size of 60 per high and low MDSC groups. (F) Survival curve of patients (top and bottom 60 patients) stratified on high (red) and low (blue) expression of the MDSC gene signature. (G) Percent of patients with *TP53* mutations (red) and no mutations (blue) in the high and low expression groups (n=60 per group). Statistical significance was determined by the log rank test and the mean as ±SEM. **H-M.** *Ex vivo* re-stimulation with PMA and ionomycin of splenocytes and dLN collected from FVB mice injected with one cell line per genotype at day 7 post tumor challenge followed by intracellular cytokine staining (ICS). Each point represents one mouse (cohorts size n=5) with the mean represented as ±SEM. (H) Flow cytometry analysis of spleens from mice bearing isogenic cell lines KC2-p53^{WT} (blue circle) and KC2-p53^{KO} (open circles) tumors stained by ICS for IFNγ. (I) Graphs show frequencies of IFNγ-producing CD4$^+$ cells in the spleen and dLN upon re-stimulation in KC1 (black) and KFC1 (red) tumor-bearing mice. (J) flow cytometry representative plot of *ex vivo* re-stimulated lymphocytes from tumor dLN from mice bearing KC1 or KFC1 tumors from each cell line showing CD44 expression against IFNγ signal. (K) Frequencies of CD8$^+$ T cell producing IFNγ upon *ex vivo* re-stimulation and ICS of splenocytes and dLN of FVB mice bearing isogenic cell lines KC2-p53^{WT} (blue) and KC2-p53^{KO} (open circle) tumors. (L) Bar graphs display frequencies of IFNγ-producing CD8$^+$ cells in the spleen and dLN upon *ex vivo* re-stimulation of KC1 and KFC1 tumor bearing FVB recipients. (M) Plots represent surface CD44 and IFNγ of gated CD8$^+$ T cells in the spleen of FVB mice harbouring KC1 or KFC1 tumors. An unpaired Student's t-test was applied where not specified. P-values range from *p<0.05, **p<0.01, ***p<0.001.

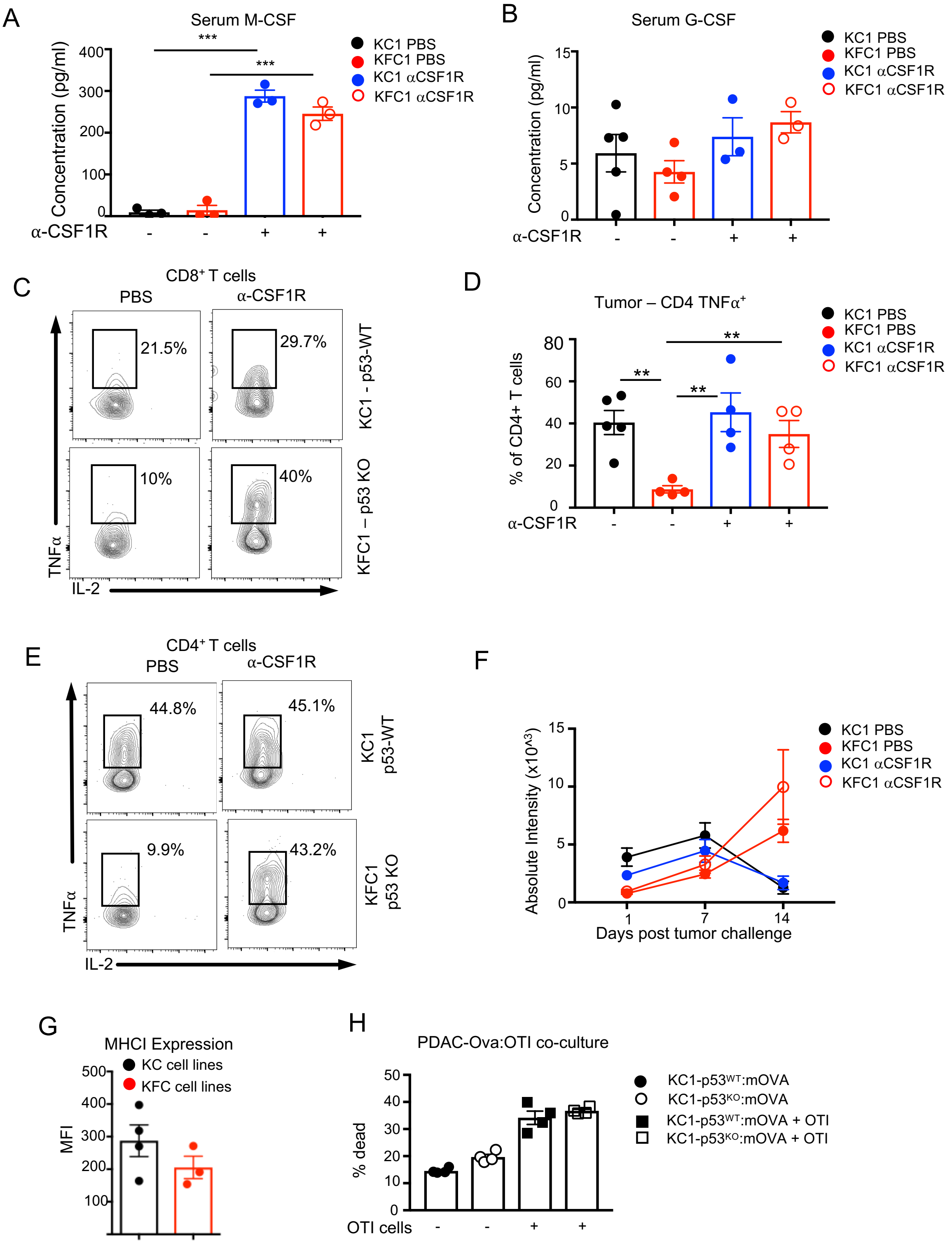

Figure S4. αCSF1-R therapy relieves T-cell suppression in p53-deleted tumors independently of tumor growth and MHCI presentation, Related to Figure 4

A-B. Serum from FVB mice injected with the KC1 and KFC1 cell lines either treated with PBS vehicle (black and red circles) or anti-CSF1-R blockade (blue and open red circles respectively) was collected and probed by ELISA for circulating (A) M-CSF and (B) G-CSF. Tukey's multiple comparison test was applied for statistical analysis and the mean is represented as ±SEM with sample sizes ranging from n=3-5. **C-E**. *Ex vivo* re-stimulation followed by ICS of tumor digests from tumors derived from the KC1

and KFC1 cell lines in FVB recipient mice treated with vehicle (PBS) or CSF1-R blockade. (C) Flow cytometry plots representing TNFα and IL2 staining of one individual tumor per cohort of infiltrating $CD8^+$ T cells. (D) Graph displays percentage of intratumoral $CD4^+$ T cells secreting TNFα from individual tumors from KC1 untreated (black), KFC1 untreated (red), KC1 treated (blue) and KFC1 treated (red open circles) tumor-bearing mice. Cohorts range from n=4-5 and the mean is represented as ± SEM with a Tukeys multiple comparison test applied. (E) Representative flow cytometry plot of intratumoral $CD4^+$ T cells expressing TNFα and IL2 **F.** Tumor growth kinetics show absolute fluorescence intensity of iRFP signal measured over 14 days by the Pearl imager with cohort sizes of n=5 per condition and the mean is represented as ±SEM. **G.** MHCI expression of 3 individual KFC and KC tumor-derived cell lines analysed by flow cytometry with the mean as ±SEM. Graph represents one of three independent experiments and an unpaired Student's t test was applied. **H.** PDAC isogenic cells ectopically expressing model antigen, ovalbumin, by retroviral introduction into KC1-$p53^{WT}$ (KC1-$p53^{WT}$:mOVA – black circles) and KC1-$p53^{KO}$ (KC1-$p53^{KO}$:mOVA – open circles). Pre-activated OTI cells with SIINFEKL peptide were co-cultured with isogenic cells expressing OVA (KC1-$p53^{WT}$:mOVA – black box and KC1-$p53^{KO}$:mOVA - open box). Cell viability was measured by propidium iodide (PI) staining and graphs show percentage of dead cells marked as PI^{+ve}. The mean is represented as ± SD and the graph represents one of three independent experiments. P values range from *p<0.05, **p<0.01, and ***p<0.001.

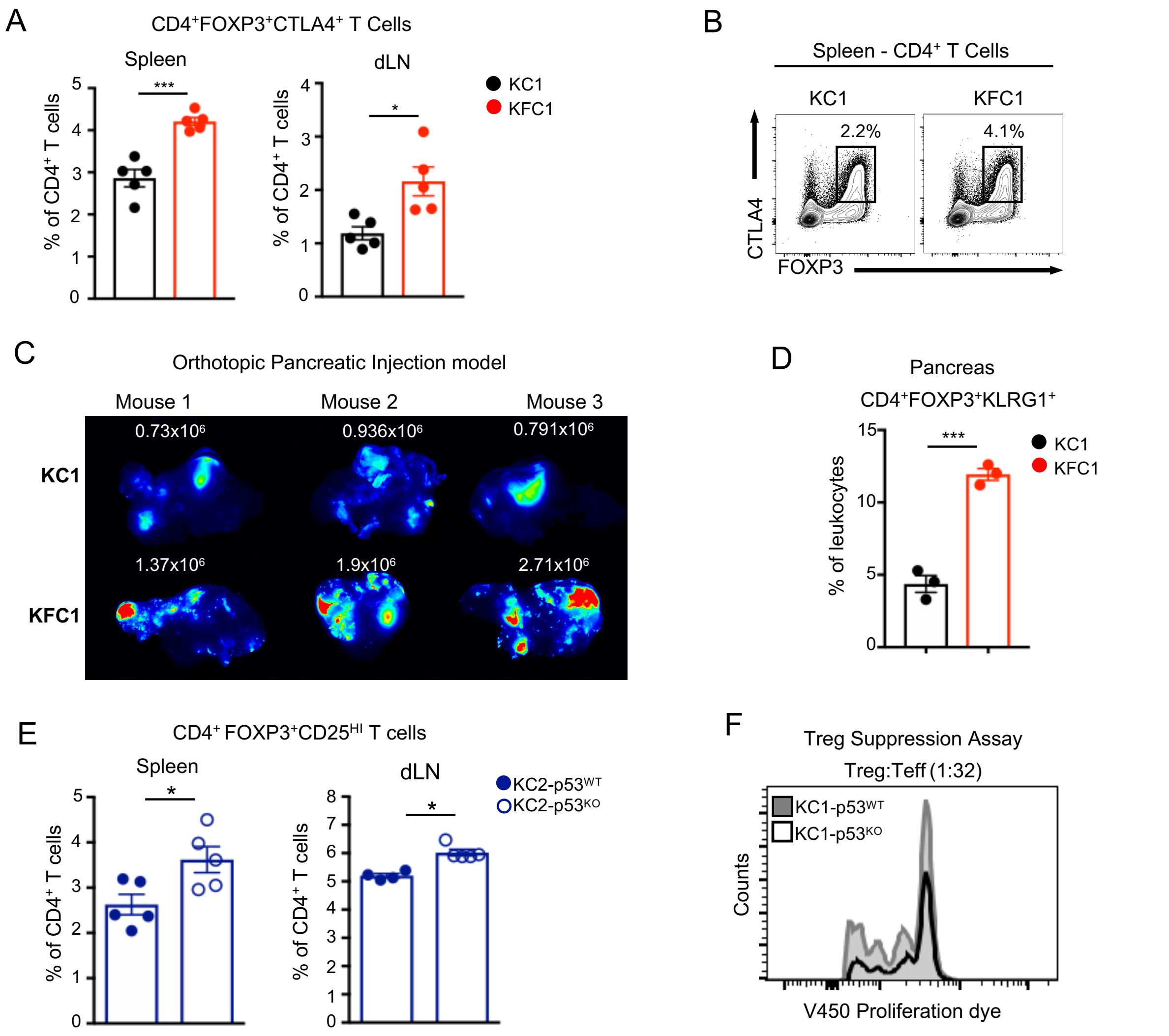

Figure S5. Cancer-specific loss of p53 increases intra-tumoral and systemic regulatory T cells, Related to Figure 5

A-B. Flow cytometry analysis for CD4$^+$FOXP3$^+$CTLA-4$^+$ Treg intracellular staining of splenocytes and dLN at 7 days of FVB mice injected with the KC1 (black) or KFC1 (red) cell lines (n=5) and the mean represented as ±SEM. (A) lymphocytes were gated on CD4$^+$ T cells and plotted for CTLA-4 and FOXP3 co-expression in the spleen and dLN. (B) Representative plot of one KC1 spleen and one KFC1 spleen gated on CD4$^+$ T cell populations and analyzed for CTLA-4 and FOXP3 co-expression. **C.** iRFP fluorescence analyzed *ex vivo* by the Pearl imager of orthotopic pancreatic injections into 3 FVB mice with the KC1 and KFC1 PDAC-derived cell lines 7 days post-surgery. **D.** Pancreatic infiltrating leukocytes gated on CD4$^+$T cells and analysed for CD4 and FOXP3 expression. The mean is represented as ±SEM. **E.** Surface and intracellular expression of CD25^{hi} FOXP3$^+$ of splenic and lymphoid CD4$^+$ T cells in KC2-p53^{WT} (blue) and KC2-p53^{KO} FVB recipients (open). Cohort sizes were n=5 and the mean is represented as ±SEM. **F.** CD4$^+$CD25$^+$ T cells isolated and sorted from KC1- p53^{WT} and KC1-p53^{KO} tumor-bearing FVB mice and co-cultured with anti-CD3 (1µg/ml) and CD4$^+$CD25$^-$ T cells (Teff) stained with V450 proliferation dye. Representative flow cytometry plot of Teff proliferation co-cultured with KC1- p53^{WT} Treg (grey shaded) and KC1-p53^{KO} Tregs (black line) at a Treg:Teff ratio of 1:32. Unpaired Student's t-tests were performed and p values are *p<0.05, **p<0.01, ***p<0.001.

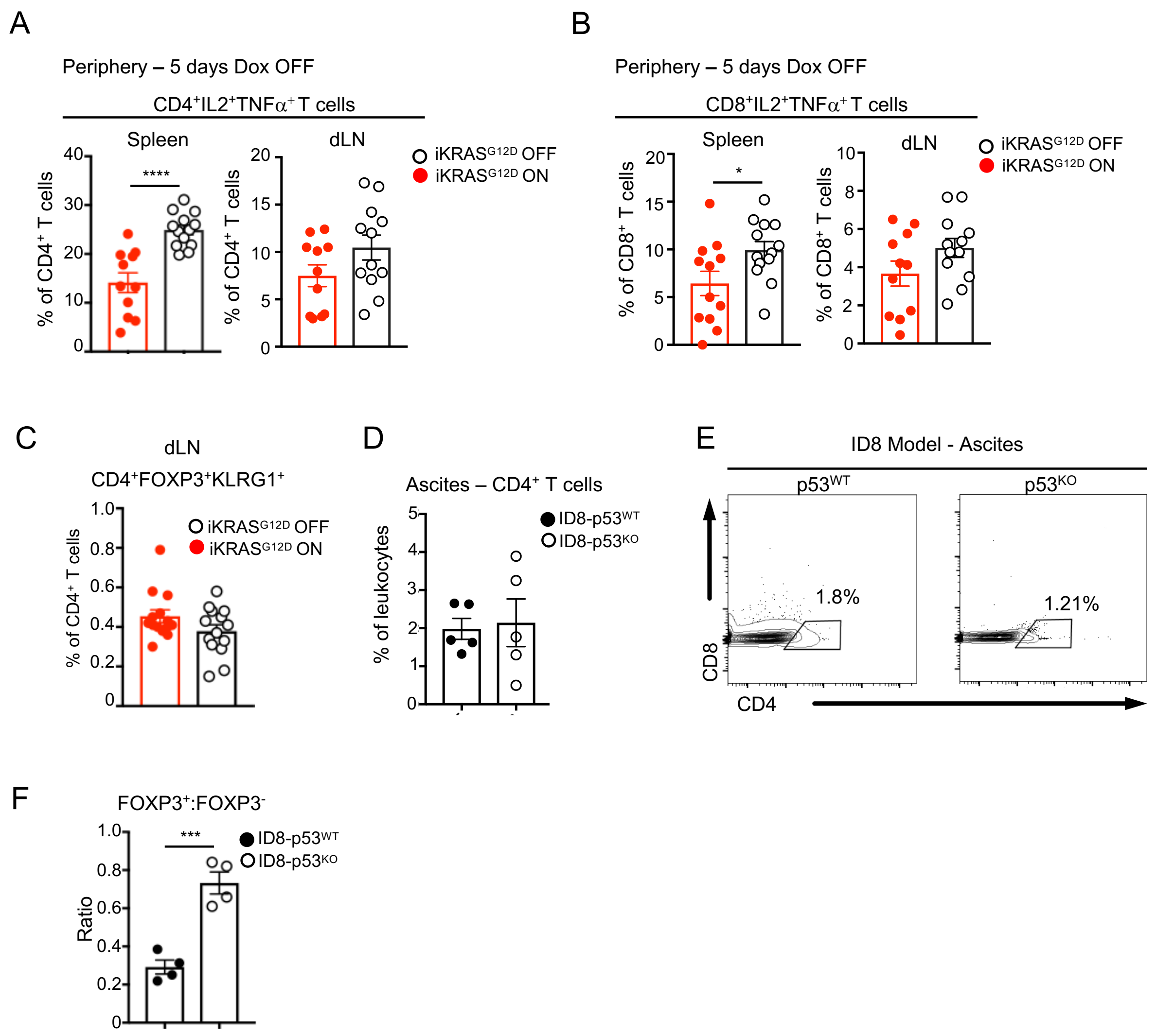

Figure S6. *Kras* mutations coordinate with p53 loss for suppressing Teffector responses, but not Treg accumulation, Related to Figure 6

A-C. Flow cytometry analysis at 5 days Dox OFF (open) and Dox ON (red) after 7 days post injection of iKRAS cell lines into FVB recipient mice. Cohort sizes were n=12-13 and each dot represents a single mouse with the mean represented as ± SEM. (A) Flow cytometry analysis of $CD4^+$ T cells of the spleen and dLN producing IL2 and TNFα upon *ex vivo* re-stimulation. (B) Flow cytometry analysis of $CD8^+$ T cells of the spleen and dLN producing IL2 and TNFα upon *ex vivo* re-stimulation. (C) Flow cytometry analysis of $CD4^+$ T cell populations expressing FOXP3 and KLRG1 in the dLN of FVB tumor bearing mice. **D-F.** Ascites of ID8 syngeneic (C57Bl6/J) model of ID8-$p53^{WT}$ (black) and ID8-$p53^{KO}$ (open circles) were analyzed by flow cytometry with sample sizes ranging from n=4-5 and the mean represented as ± SEM. (D) Graph shows $CD4^+$T cell frequencies of individual mice and the mean as ± SEM with a cohort size of n=5. (E) Flow cytometry representative plot for surface expression of CD4 (X-axis) and CD8 (Y-axis). (F) Graph shows the ratio of $FOXP3^+$ versus negative cells within the $CD4^+$ population in the ascites of ID8-$p53^{WT}$ (black) and ID8-$p53^{KO}$ donor cells (open circles). The mean is represented as ±SEM, n=4. Unpaired Student's t-test were performed for each graph. P-values range from *p<0.05, **p<0.01, ***p<0.001.
